# Isoniazid monotherapy for tuberculosis prevention during pregnancy and adverse outcomes in women living with HIV in Uganda: A propensity score–weighted analysis of routine care data

**DOI:** 10.1371/journal.pgph.0005829

**Published:** 2026-09-25

**Authors:** Joseph Musaazi, Christine Sekaggya-Wiltshire, Stella Zawedde-Muyanja, Proscovia M. Namuwenge, M. Sanni Ali, Yukari C. Manabe, Barbara Castelnuovo, Nele Brusselaers

**Affiliations:** 1 Infectious Diseases Institute, School of Medicine, College of Health Sciences, Makerere University, Kampala, Uganda; 2 Global Health Institute, Faculty of Medicine and Health Science, University of Antwerp, Antwerp, Belgium; 3 School of Medicine, University of St Andrews, St Andrews, Scotland, United Kingdom; 4 National Tuberculosis and Leprosy Program, Uganda Ministry of Health, Kampala, Uganda; 5 Department of Disease Control, LSHTM, London, United Kingdom; 6 VIBRANIUM RESOURCES Foundation, Addis Ababa, Ethiopia; 7 Division of Infectious Diseases, Department of Medicine, Johns Hopkins University School of Medicine, Baltimore, Maryland, United States of America; 8 Department of Women’s and Children’s Health, Karolinska Institutet, Stockholm, Sweden; JIPMER PSM: Jawaharlal Institute of Post Graduate Medical Education and Research Department of Preventive and Social Medicine, INDIA

## Abstract

Data on the safety of tuberculosis preventive treatment (TPT) during pregnancy particularly from routine care settings in high tuberculosis (TB) burden countries remain limited. We evaluated the association between TPT exposure during pregnancy and adverse pregnancy outcomes among pregnant women living with HIV (WLHIV) in Uganda. We conducted a retrospective cohort study using routinely collected data from five public urban primary health facilities in Kampala, Uganda. We included pregnant women living with HIV on antiretroviral therapy (ART) between 2016 and 2022. The primary outcome was a composite of adverse pregnancy outcomes: miscarriage, stillbirth, low birth weight, neonatal complications, or maternal/neonatal death. A secondary composite outcome excluded neonatal and maternal death. There was no preterm delivery documented in this sample. The primary exposure was 6-month isoniazid preventive therapy (IPT) during pregnancy. We applied inverse probability of treatment weighting (IPTW) using propensity scores from logistic regression to adjust for confounding. Missing data on covariates and outcome were addressed using multiple imputation. Analysis was performed using R software v.4.3.3. We analyzed data from 521 pregnant WLHIV, the median age was 28 years (interquartile range 24–31), 44% of whom received IPT during pregnancy. Overall, 10.0% experienced an adverse pregnancy outcome. No significant differences were observed between IPT-exposed and unexposed groups for the primary (10.3% vs. 9.6%; p = 0.81) or secondary (10.3% vs. 8.5%; p = 0.50) composite outcomes. IPTW-adjusted analysis showed no significant association between IPT exposure and adverse pregnancy outcomes (weighted OR: 1.06; 95% CI: 0.68–1.63; p = 0.81). Sensitivity and subgroup analyses yielded consistent findings. Exposure to 6-month isoniazid TPT during pregnancy was not statistically associated with adverse pregnancy outcomes among women on ART in routine care settings. Strengthened pharmacovigilance and systematic clinical reporting are essential to ensure maternal and neonatal safety as TPT coverage expands in high TB/HIV burden settings.

## Introduction

The World Health Organization (WHO) recommends tuberculosis preventive treatment (TPT) for people living with HIV (PLHV), including pregnant women, to reduce progression to active tuberculosis (TB) [1].However, pregnancy may increase susceptibility to hepatoxicity among women living with HIV [2], with evidence suggesting a higher risk among those exposed to isoniazid preventive therapy (IPT) compared with unexposed women [3]. Concerns about the safety of IPT during pregnancy were heightened by findings from the IMPAACT P1078 randomized controlled trial, conducted across 13 sites in eight high TB-burden countries. The trial reported a higher frequency of adverse pregnancy outcomes among women who initiated IPT during pregnancy (24%) compared with those who deferred initiation to the postpartum period (17%) [4]. Reported outcomes included stillbirth, spontaneous abortion, low birth weight, preterm delivery, and congenital anomalies.

In contrast, a systematic review that included the IMPAACT P1078 trial reported mixed findings. Of the five studies reviewed, only the P1078 trial demonstrated a significant increase in adverse pregnancy outcomes associated with IPT, while the remaining observational studies reported no association [3]. These divergent findings highlight uncertainty about the safety of IPT during pregnancy, particularly in routine programmatic settings where populations and clinical monitoring differ from trial conditions.

Uganda bears a substantial dual burden of TB and HIV. HIV prevalence among Ugandan women aged 15–49 years was 6.6% in 2022, nearly double that of men (3.6%) [5], and remained similarly higher among women in 2024 (6.4% versus 3.4%) [6]. The 2015 Uganda National TB Survey estimated TB incidence at 198 cases per 100,000 population [7]. In this high-burden context, TB occurring during pregnancy or the postpartum period poses particular risks and has been consistently associated with serious adverse outcomes, including spontaneous abortion, low birth weight, preterm delivery [8], and perinatal mortality [9].

In 2015, the Uganda Ministry of Health (MoH) adopted the WHO recommendation to provide TPT to all people at risk of TB. This includes all PLHV without active TB and household contacts of confirmed TB cases. From 2015 until 2021, isoniazid monotherapy for six months was the only TPT regimen [10]. Between 2018 and 2023, Uganda intensified TPT coverage through strategic interventions. These included a 100-day accelerated IPT scale-up campaign launched in July 2019, targeting 300,000 PLHIV with completion rates above 90% [11]. In 2021, Uganda updated national guidelines to integrate TPT into routine HIV care for all PLHV without TB symptoms [12]. The revised guidelines endorsed shorter rifamycin-based TPT regimens alongside isoniazid monotherapy to improve adherence. Earlier guidelines allowed isoniazid use at any stage of pregnancy. However, the 2021 revision introduced restrictive criteria: TPT during pregnancy was recommended only for women with CD4 counts <200 cells/mm^3^, WHO stage III or IV HIV disease, or recent TB exposure. For others, initiation was deferred until three months postpartum [12].

By 2023, TPT uptake and completion among PLHIV in Uganda had exceeded 90%, with higher completion among women than men [13,14]. This reflects strong program implementation and national commitment to TB control. Despite these gains, data on TPT safety during pregnancy remain limited, particularly from routine care settings in high-burden countries. While both clinical trials and observational studies have assessed safety [3,4,15,16], evidence from real-world programmatic data remains scarce. Although P1078 trial demonstrated a modest increase in adverse outcomes with pregnancy-initiated IPT, WHO and Uganda MoH recommend TPT for pregnant WLHIV in high TB burden settings, emphasizing that benefits outweigh risks [10,17,18]. This recommendation reflects the substantially elevated risk of progression to active TB among pregnant women with HIV and the severe consequences of untreated TB for both mother and infant [18].

This study aimed to evaluate the effect of the isoniazid monotherapy TPT use during pregnancy on adverse outcomes, including spontaneous abortion or miscarriage, stillbirth, low birth weight, preterm delivery, congenital anomalies, neonatal complications, and maternal or neonatal death. Using routinely collected programmatic data, we assessed both the prevalence of adverse outcomes and the potential effect of TPT exposure during pregnancy.

## Materials and methods

### Study design and setting

We conducted a retrospective cohort study using routinely collected clinical data from five public primary health care facilities in Kampala, Uganda. The clinics provide free HIV care and treatment through the President’s Emergency Plan for AIDS Relief (PEPFAR) program, including antiretroviral treatment, and prophylaxis for opportunistic infections and tuberculosis, and antenatal services. Clinical data are captured in paper-based registers and the Uganda electronic medical record (EMR) system.

The study sites were purposively selected due to the relative completeness and reliability of their clinical documentation compared to other facilities in the region. Kampala was selected as the study setting due to its high burden of both HIV and tuberculosis (TB) relative to other regions in Uganda.

### Study population

The study included pregnant WLHIV, aged ≥15 years, who initiated antiretroviral therapy (ART) between January 2016 and December 2022. This period coincided with the rollout of the HIV Test- and-Treat strategy and expanded TPT provision in Uganda [19]. We included women who were already on ART or initiated on ART during the index pregnancy. We excluded women with unknown ART status, those with a ≥ 12-month gap in HIV clinic attendance, or those who initiated TPT before ART to minimize confounding.

### Sample size and statistical power

We estimated a sample size of 538 pregnant WLHIV (269 per group) to achieve 80% power to detect a ≥ 10 percentage-point difference in adverse pregnancy outcomes between TPT-exposed and unexposed groups, assuming a 17% baseline risk [4], with a two-sided alpha of 0.05 using Pearson’s Chi-square test. However, the final sample size was 521 women due to missing patient files and registers at some study facilities.

### Participants’ selection and sampling strategy

Using the Uganda EMR system, we generated a sampling frame of eligible WLHIV with documented pregnancy. Participants were proportionally allocated across the five sites and selected using systematic random sampling, stratified by TPT-exposed. The sampling interval was calculated by dividing the total number of eligible women (sampling frame) at each site by the number of participants allocated to that site.

Despite available EMR data, sampling was required because pregnancy variables and outcomes were inconsistently recorded in EMRs; targeted extraction from paper registers ensured complete, consistent capture.

### Data extraction and collection

Between July 2020 and March 2021, trained research assistants abstracted demographic, clinical, and pregnancy data from paper-based registers; TPT, maternity and antenatal registers using a structured abstraction tool programmed in Open Data Kit (ODK) [20]. Built-in validation checks and daily reviews of collected data ensured quality. Updated data were extracted from the Uganda EMR in December 2023. Additional data were obtained directly from the Uganda Electronic medical records (EMR) database system [21] using a pre-defined abstraction guide. Data from multiple sources were linked using unique patient HIV clinic identifiers and de-identified before analysis.

### Outcome measures

The primary outcome was a composite measure of adverse pregnancy outcomes, defined as the occurrence of any of the following: spontaneous abortion/miscarriage, stillbirth, low birth weight, neonatal complications, neonatal death, or maternal death. A secondary composite pregnancy outcome excluded neonatal and maternal death to focus on adverse fetal outcomes; to align with definitions used in published literature [16]. Individual pregnancy outcomes were assessed as secondary outcomes.

For women with multiple pregnancies, the most recent pregnancy was analysed. Pregnancy onset was defined as the date of the first clinic visit at which pregnancy was documented, which served as the baseline for follow-up until the occurrence of the pregnancy outcome (as defined in the preceding section) or until 28 days for neonatal death or 42 days for maternal death. Preterm birth was excluded from the composite outcome due to a lack of documented cases in the reviewed pregnancy records.

### Pregnancy outcome ascertainment

Pregnancy outcomes were primarily ascertained through review of maternity and postnatal paper-based registers, which routinely record delivery details and key maternal and neonatal outcomes. Documentation of pregnancy outcomes within the HIV clinic module was limited. Ascertainment of outcomes relied solely on clinical documentation in delivery or postnatal records, as there is no established surveillance system in most public health facilities in this setting.

### Definitions of individual pregnancy outcomes

Pregnancy outcomes were defined as follows. Spontaneous abortion refers to the loss of a fetus before 20 weeks of gestation, while stillbirth is the death of a fetus at or beyond 20 weeks. Low birth weight describes infants born weighing less than 2,500 grams (5 pounds, 8 ounces). Neonatal mortality is defined as the death of a live-born infant within the first 28 days of life. Maternal mortality refers to the death of a woman during pregnancy, childbirth, or within 42 days of termination, due to causes related to or aggravated by the pregnancy or its management. Perinatal mortality refers to the death of a fetus or newborn occurring around the time of birth, combining stillbirths and early neonatal deaths. Neonatal complications at birth refer to conditions in the immediate postnatal period indicating impaired adaptation, including failure to initiate breathing requiring stimulation or resuscitation and other acute conditions needing clinical management.

### Exposure

The primary exposure was taking tuberculosis preventive therapy (TPT) using 6-months isoniazid monotherapy during pregnancy, which was the only regimen available during the study period. TPT exposed during pregnancy implies that a woman received TPT either as a continuing course or through initiation during pregnancy. A secondary analysis classified TPT exposure by trimester: no exposure, 1st trimester (<13 weeks), 2nd trimester (13–28 weeks), and 3rd trimester (29–40 weeks), in accordance with national guidelines [22] and previous studies [4,16]. TPT exposure was additionally categorized using 3 levels to examine whether the risk of adverse pregnancy outcome differ across: never exposed to TPT, pre-pregnancy TPT completion, and TPT exposure during pregnancy. TPT exposure was defined based on the timing of initiation rather than completion, due to incomplete documentation of treatment completion in the paper-based TPT register. Therefore, women who initiated TPT before experiencing an adverse pregnancy outcome were considered exposed. This approach is consistent with an intention-to-treat analytical framework.

### Covariates

Potential covariates included both clinical and reproductive factors. Clinical variables comprised viral load (copies/mL), nadir CD4 count at ART start (cells/μL), ART regimen type at the time of pregnancy, ART duration in months, WHO HIV clinical stage (I/II vs III/IV), body mass index (BMI, kg/m^2^) and, ART initiation calendar year before and after September 2018 to coincides with dolutegravir-based ART regimen roll-out in Uganda. Viral load during pregnancy was defined as the closest measurement within 12 months before or 6 months after the pregnancy event date. Baseline CD4 count at ART start was defined as any test conducted within 6 months before or 3 months after ART start. Reproductive factors included maternal age (in years), gestational age (in weeks) at first antenatal care (ANC) visit, parity, gravidity, number of ANC visits, and anemia. Information on other maternal health conditions such as preeclampsia, hypertension, diabetes, and sexually transmitted infections (including syphilis) were limited or inconsistently recorded and therefore excluded from the analysis. Pre-pregnancy weight was defined as the most recent recorded weight within six months prior to the first documented pregnancy visit.

### Statistical analysis

Descriptive statistics (frequencies, percentages, means with standard deviations, or medians with interquartile ranges) were used to summarize participant characteristics. For baseline weight and other continuous covariates, missing values were replaced using the most recent measurement recorded before study entry (last observations carried forward, LOCF). Because these measurements were obtained during routine clinical follow-up and were generally recorded close to the baseline date, they were considered reasonable proxies for missing baseline values.

Adverse pregnancy outcomes were compared between exposure groups using Pearson’s Chi-square test. To adjust for confounding, we applied inverse probability of treatment weighting (IPTW) with stabilized propensity scores, followed by weighted logistic regression.

Missing data on covariates and outcome were handled using multiple imputation by chained equations (MICE). Initially a reasonable number, 10 imputations were performed, and then increased to 20 and 30 imputations, numbers close to proportion of high missingness of variable, and checked whether estimates changed materially. All variables included in the analytical model, including the outcome, exposure, and propensity score covariates, were included in the imputation model. Binary variables were imputed using logistic regression, unordered categorical variables using polytomous regression, and continuous variables using predictive mean matching (S1 Table). More details of imputation models are provided in supplementary information (S1 Table).

Propensity scores were estimated separately within each imputed dataset using logistic regression. Propensity score model included maternal age, body mass index (BMI), haemoglobin level, ART duration, CD4 count, viral load status, ART initiation period, parity, and health facility. Stabilized inverse-probability-of-treatment weights were then calculated within each imputed dataset. Weighted logistic regression models were fitted separately in each completed dataset, and regression coefficients and standard errors were combined across imputations using Rubin’s rules implemented through the mice package. We additionally calculated the effective sample size (ESS) after weighting to assess weight variability and extreme weights. The effective sample size (ESS) was calculated for each imputed dataset as a fraction of squared sum of weights divided by sum of squared weights. The ESS quantifies the amount of information retained after weighting and provides an assessment of weight variability.

Covariate balance between TPT‑exposed and unexposed women was assessed using standardized mean differences (SMDs). Balance was evaluated before and after inverse probability of treatment weighting, with SMDs < 0.10 considered indicative of adequate balance. The distribution of stabilized weights was examined using summary statistics and graphical diagnostics to assess the presence of extreme weights.

Subgroup analyses were performed to examine effect modification by CD4 count (<200 vs ≥ 200 cells/μL) at ART initiation an indicator of advanced HIV disease, viral load (<200 vs ≥ 200 copies/mL) reflecting viral suppression per 2022 Uganda Consolidated HIV/AIDS Guidelines [23], age (<25 vs ≥ 25 years), ART initiation year (<2018 vs ≥ 2018) as a proxy for DTG-based ART roll-out, and BMI (<18.5, 18.5– < 25, ≥ 25 kg/m^2^) representing underweight or overweight status. These variables were selected based on data availability, and completeness in routine HIV care database at the health facilities studied.

Sensitivity analyses included unadjusted, covariate-adjusted, and Firth penalized logistic regression models. The latter used to address sparse outcomes. Firth regression was not used with multiple imputation due to incompatibility with imputed datasets. Statistical significance was set at 5% level. Reporting follows Strengthening the Reporting of Observational Studies in Epidemiology (STROBE) guidelines (S1 File STROBE Checklist).

Data were cleaned using STATA v.17.0 (Stata Corp, College Station, TX, USA), and analysis performed using R software v.4.3.3 (R Computing, Vienna, Austria).

### Ethics approval and consent to participate

The AIDS Support Organization Research Ethics Committee (TASOREC), Kampala, Uganda (number: TASOREC/085/19-UG-REC-009) and the Uganda National Council for Science and Technology, Kampala, Uganda (UNCST number: HS729ES) granted ethical approval for the study. Due to the retrospective study design and that this was a public health surveillance, the need for patient informed consent was waived by the Ethic Committee.

## Results

### Participants description

Between 2016 and 2022, a total of 13,401 pregnant women living with HIV were recorded in HIV care clinics across the five public primary health care facilities. From this population, 538 women were sampled. Of these, 521 were identified through maternity and postnatal paper-based registers and were included in the final analysis (Table 1). Among the 521 included women, 227 (44%) had used IPT for TPT during pregnancy, while 294 (56%) had not. Among those who used IPT, 139 (61%) started IPT in 1^st^ trimester, 58 (26%) in the 2nd trimester, and 30 (13%) in the 3rd trimester. In the IPT unexposed group, 149 (51%) had completed IPT course before the onset of pregnancy, and 145 (49%) had never received IPT.

**Table 1:** Pregnant women living with HIV’ characteristics at first antenatal visit by TPT exposure status during pregnancy*.

Characteristics	Total	TPT Unexposed group	TPT Exposed group	P-value
	N = 521	N = 294	N = 227	
Age in years, median (IQR) †	28 (24,31)	28 (24,32)	28 (24,31)	0.403‡
Parity ≥ 2 births†	238 (50.3)	125 (48.4)	113 (52.6)	0.374
BMI in Kgs/m^2^, median (IQR) †	24.9 (22.3, 27.8)	24.9 (22.2, 27.8)	24.9 (22.7, 27.5)	0.750‡
WHO HIV stage 3/4, n (%) †	3 (0.6)	1 (0.4)	2 (0.9)	0.469§
Gestation age at first ANC visit in weeks, median (IQR)	24 (18,30)	23 (18,30)	26 (20,32)	0.013‡
CD4 count at ART start, in cells/mL, median (IQR) †	408 (243, 584)	413 (215, 586)	396 (268, 584)	0.820‡
CD4 count <200 cells/mL, n (%)	73 (18.7)	45 (21.2)	28 (15.6)	0.158
Viral load at pregnancy †				
< 200 copies/mL, n (%)	299 (89.2)	163 (88.1)	136 (90.7)	0.452
< 1000 copies/mL, n (%)	308 (91.9)	168 (90.8)	140 (93.3)	0.399
ART start after 2018 †¥	153 (30.5)	57 (20.7)	96 (42.3)	<0.001
ART duration in months, median (IQR) †	22 (0, 53)	25 (6, 56)	16 (0, 46)	<0.001‡
ART duration ≥ 2 years, n (%) †	245 (48.9)	144 (52.6)	101 (44.5)	0.072
ART regimen at pregnancy ¶				
DTG based	33 (6.7)	21 (7.7)	12 (5.4)	0.580§
EFV based	444 (89.5)	240 (87.9)	204 (91.5)	
NVP based	10 (2.0)	7 (2.6)	3 (1.3)	
ATV/r or LPV/r	9 (1.8)	5 (1.8)	4 (1.8)	

* Number of women exposed to TPT during pregnancy in: 1^st^ trimester (n = 139), 2^nd^ trimester (n = 58), and 3^rd^ trimester (n = 30). Among the unexposed group, n = 149 had completed TPT before pregnancy onset, n = 145 had never been exposed to TPT.

† Missing values: Age (n = 3), parity (n = 48), BMI (n = 38), WHO stage (n = 47), CD4 (n = 130; 25%), viral load (n = 186; 36%), and ART duration (n = 20; 4%). The 20 participants missing ART start dates were included based on documented ART regimens indicating they were already on treatment. Summary statistics (medians [IQR] and percentages) were calculated using available-case denominators (i.e., participants with non-missing data for each specific variable).

‡ P-values derived using Man-Whitney Wilcoxon rank-sum test to comparing medians across groups, else P-values were derived using Pearson Chi-square test. § Fisher’s exact p-values.

¥ ART initiation calendar year before and after September 2018 to coincides with dolutegravir-based ART regimen roll-out in Uganda. This stratification helped to explore whether there’s interaction between TPT exposure before versus after DTG-based regimen DTG roll-out in Uganda.

††Time since initiated TPT to pregnancy follow-up start among women who completed TPT prior the index pregnancy

¶ ART regimen at pregnancy, 97% were on TDF based regimen on overall (96% in unexposed group, 98% in exposed group).

Overall, the median age at current pregnancy was 28 years (interquartile range [IQR] 24–31 years), 50.3% (238/473) had ≥ 2 prior live births, 0.6% were in HIV WHO stage 3 or 4, with median time on ART of 22 months (IQR: 0–53 months). At their first recorded antenatal visits, the median gestation age was 24 weeks (IQR: 18–30 weeks), implying that majority of the women had their first ANC visit in their 2^nd^ trimester (Table 1).

### Pregnancy outcomes

Of the 521 enrolled participants, pregnancy outcome data were available for 472 (90.6%) (Table 2). No significant differences between observed and unobserved baseline characteristics (supplementary S7 Table). Among those with complete data, 47 women (10.0%) experienced at least one adverse pregnancy outcome.

**Table 2:** Pregnancy outcomes by TPT-exposure status during pregnancy.

Pregnancy outcomes¶	TPT-exposed n (%) N = 213	TPT-unexposed n (%) N = 259	Risk difference (%)	P-value
**Composite 1:** fetus death, LBW, neonatal complications at birth, neonatal death or maternal death	22/213 (10.3)	25/259 (9.6)	0.7 (-4.8,6.1)	0.81
**Composite 2:** fetus death, PTD, LBW, or neonatal complications	22/213 (10.3)	22/259 (8.5)	1.8 (-3.5,7.1)	0.50
*Specific adverse outcomes*				
Low birth weight (<2500g)	6 (2.8)	10 (3.8)	-1.0 (-4.3, 2.2)	0.53
Spontaneous abortion or miscarriage	2 (0.9)	6 (2.3)	-1.4 (-3.6, 0.9)	0.30†
Stillbirth	7 (3.3)	2 (0.8)	2.5 (-0.1, 5.1)	0.09†
Neonatal death	0	1 (0.4)	-0.4 (-1.1, 0.4)	0.99†
Maternal death	0	2 (0.8)	-0.8 (-1.8, 0.3)	0.50†
Neonatal complication at birth: Difficulty in breathing†	7 (3.3)	4 (1.5)	1.7 (-1.1, 4.6)	0.24†

¶ Analysis performed on n = 472 women who had pregnancy outcomes, 49/521 (9%) had missing pregnancy outcome.

†Fisher’s exact p-value comparing adverse pregnancy outcomes between women exposed to TPT during pregnancy versus those who were not; where expected cell count is less than 5. Else, Pearson Chi-square p-values are presented. % denote column percentages.

Composite 1 includes all adverse pregnancy outcomes; fetus death (spontaneous abortion/miscarriage or stillbirth), LBW (low birth weight < 2500g or 5.51 pounds), neonatal complication, neonatal death (<28 days) or maternal death (<42 days).

Composite 2 includes only outcomes of the ongoing pregnancy; fetus death (spontaneous abortion/miscarriage or stillbirth), LBW, or neonatal complications [16]. There was no preterm delivery or congenital anomaly documented in this sample.

† Difficulty in breathing includes newborn who did not initiate spontaneous breathing at birth and required stimulation and/or resuscitation (e.g., bag-and-mask ventilation).

There was a slightly higher prevalence of adverse pregnancy outcomes among women exposed to TPT during pregnancy compared to those unexposed (22/213, 10.3% vs. 25/259, 9.6%), though the difference was not statistically significant (p = 0.81). Similarly, no significant difference was observed for the secondary composite outcome which focused on adverse fetal outcomes (10.3% vs. 8.5%; p = 0.50). After adjusting for confounding using IPTW and multiple imputation of missing data, the pooled weighted analysis showed no significant association between TPT exposure and adverse pregnancy outcomes (pooled weighted odds ratio [OR], 1.06; 95% confidence interval [CI], 0.68 to 1.63; p = 0.81) (Table 3). Standardized mean differences indicated notable covariate imbalance before weighting. After stabilized IPTW, all covariates achieved acceptable balance (absolute SMD < 0.10), demonstrating effective mitigation of measured confounding (S2 Table and S1 Fig). The stabilized IPTW weights shows that most weights clustered around 1, with a modest right skewed tail (S2 Fig). Across imputed datasets, the effective sample size ranged from 382-405 (median 397), representing 81–86% of the original sample (N = 472), indicating stable weights and minimal efficiency loss. The absence of extreme weight values suggests adequate covariate overlap and stable estimation of the weighted treatment effect. The distribution of composite adverse outcomes across covariates is presented in supporting information (S3 Table).

**Table 3:** Weighted odds ratios and 95% confidence intervals for the impact of Isoniazid TPT monotherapy taken during pregnancy on adverse pregnancy outcomes.

	Odds Ratio (95% CI)	*P*-value
***Primary analysis***		
IPTW weighted Logistic regression model¥	1.06 (0.68, 1.63)	0.81
***Sensitivity analyses***		
Unadjusted Logistic regression model (complete case)^¶^	1.08 (0.59, 1.97)	0.81
Unadjusted Logistic regression model (imputed)	0.96 (0.53, 1.76)	0.90
Covariates-adjusted Logistic regression model^†^	0.90 (0.45, 1.77)	0.75
Firth penalized logistic regression model^‡¶^	1.08 (0.59, 1.96)	0.80

OR = odds ratio; IPTW = inverse probability of treatment weighting; TPT = tuberculosis preventive therapy.

¥ Pooled Odds Ratios and their 95% confidence intervals (95%CI) were estimated from Pooled Weighted Logistic Regression. Propensity scores used to generate Inverse Probability Treatment weights (IPTW) were calculated using a logistic regression model with covariates: healthy facility (study site), maternal age, body mass index, anemia during antenatal care, ART duration, CD4 < 200, viral load <200, ART regimen, calendar year of ART start (pre/post-September 2018), and parity ≥2. Missing data were completed using Multiple Imputation with chained equations using 10 imputations, via predictive mean matching for continuous and logistic model for binary variables. See supporting information S1 Table for details on specific imputation models used.

¶ Complete case estimates analyzed on 472 participants (excluding 49 with missing pregnancy outcomes)

†Covariate-adjusted logistic regression model included the same variables as the PS model.

‡Firth penalized logistic regression model used to reduce sparse data bias.

Adverse pregnancy outcomes defined as proportion of composite outcome that includes any of the following: spontaneous abortion/miscarriage, stillbirth, low birth weight, neonatal complications, neonatal death, or maternal death. No preterm delivery or congenital anomaly documented in this sample.

Sensitivity analyses yielded consistent results; re-running the analysis using Firth penalized logistic regression which accounts for sparse data bias, it further revealed no effect of TPT exposure during pregnancy on odds of adverse pregnancy outcome (adjusted OR, 1.08; 95% CI, 0.59 to 1.97). Similarly, conventional complete case analysis and covariate-adjusted logistic regression with multiply-imputed data showed no significant association (Table 3). Results also remained largely unchanged when IPTW analyses were repeated using 20 and 30 imputed datasets (S6 Table).

Low birth weight was the most common adverse outcome, occurring in 16 of 472 participants (3.4%), with no significant difference between TPT-exposed and unexposed groups (p = 0.53). The overall median birth weight was 3,200 g (IQR: 3,000–3,500 g). Additionally, no significant associations were found between TPT exposure and other individual outcomes, including miscarriage, maternal or neonatal death (all p ≥ 0.20) and stillbirth (p = 0.05) (Table 2). No cases of preterm delivery and congenital anomalies were documented in the cohort.

Among women who received TPT during pregnancy, the highest prevalence of adverse outcomes was observed in those who initiated TPT in the third trimester (6/29; 20.7%), compared with those who initiated in the first (12/130; 9.2%) or second trimester (4/54; 7.4%). However, this difference did not reach statistical significance (p-value = 0.38). Although the numbers were small, stillbirth was the most frequently observed adverse event among those who initiated TPT in the third trimester (4/29, 13.8%) (S4 Table). No significant difference in composite adverse pregnancy outcomes was observed across the three TPT exposure categories (never exposed, TPT completed before pregnancy, and TPT during pregnancy; p = 0.781) (S5 Table).

**Fig 1:**
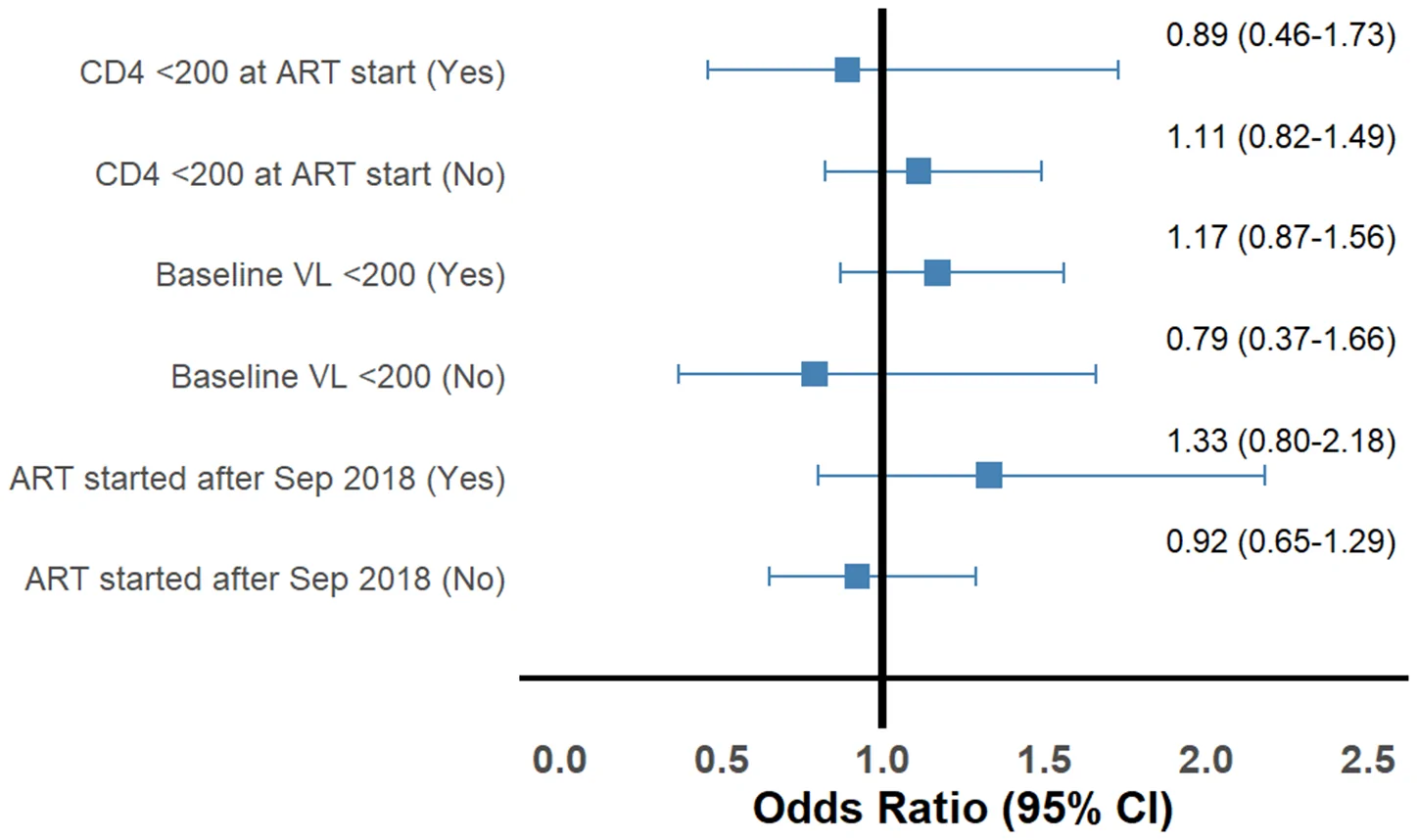
Subgroup Analysis for the impact of TPT-exposure during pregnancy on pregnancy outcome. Legend: Subgroups for ART started after Sep 2018 (Yes) denotes participants initiated on ART after September 2018, which coincides with dolutegravir-based ART regimen roll-out in Uganda, whereas ART started after Sep 2018 (No) denotes those were initiated on ART before September 2018.

In exploratory subgroup analyses (Fig 1), there was no significant interactions between TPT exposure during pregnancy and selected covariates which included; timing of ART initiation, HIV viral load at the first antenatal visit, or baseline CD4 count (all interaction p > 0.10).

## Discussion

This study contributes to the body of knowledge about safety of TPT during pregnancy. Using routinely collected data from urban HIV clinics in Uganda, we evaluated the association of isoniazid monotherapy TPT and adverse pregnancy outcomes among women on ART. Although TPT-exposed women had a slightly higher risk of adverse outcomes including miscarriage, stillbirth, low birth weight, neonatal complications, maternal, or neonatal death, these differences were not statistically significant for either composite or individual outcomes. Results were consistent for a secondary composite focused on adverse fetal outcomes (miscarriage, stillbirth, low birth weight) and remained robust across causal inference models and sensitivity analyses.

We found no evidence of effect modification by maternal viral load, baseline CD4 count, or timing of IPT initiation relative to the rollout of dolutegravir-based ART. However, the study may have been underpowered to detect interaction effects, limiting assessment of heterogeneity in IPT safety across subgroups.

Low birth weight was the most frequent adverse outcome, aligning with findings from the IMPAACT P1078 trial, which linked low birth weight to maternal factors such as poor nutrition and smoking [16]. Our dataset lacked these variables, limiting exploration of their role. Although the numbers were small, we also observed high proportions of stillbirth and neonatal complications among IPT-exposed women in the first trimester, a critical period for fetal development [24]. These signals underscore the need for pharmacovigilance and larger cohort studies to assess the safety of IPT during early pregnancy [25].

Adverse outcomes were more frequent among women initiating IPT in the third trimester. Late initiation may reflect delayed antenatal care, a known risk factor for poor outcomes such as stillbirth and preterm birth [26,27]. These findings highlight the importance of promoting early antenatal attendance and timely initiation of preventive therapies aimed to improve outcomes.

Our results align with observational studies from sub-Saharan Africa [15,28,29], and a systematic review by Hamada et al. [3], reported no increased risk of adverse pregnancy outcomes with IPT. For instance, Taylor et al. [28] found no association between IPT and adverse outcomes in Botswana, while Salazar-Austin et al. [29] and Kalk et al. [15] reported lower risks of adverse outcomes among IPT recipients in South Africa. Although these studies may be subject to residual confounding due to limited adjustment for key maternal risk factors, the consistency of findings across programmatic settings is notable. Taken together, this body of evidence including our findings supports the WHO guidance indicating that pregnancy alone should not preclude women living with HIV from receiving TPT using TB medications that are widely regarded as safe in pregnancy, including isoniazid and rifampicin [30].

In contrast, Theron et al. [16], using data from the IMPAACT P1078 trial, reported significantly higher rates of composite adverse pregnancy outcomes among women initiating IPT during pregnancy versus postpartum. This study had strong internal validity, with a large sample size (925 mother–infant pairs), conducted in 8 high TB-burden countries, and rigorous adjustment for confounders. Although we applied propensity score–weighted methods to minimize bias, residual confounding from unmeasured factors such as syphilis, hepatitis co-infection, multiple gestation, and other comorbidities may still have influenced our findings. Although non-significant, our results showed slightly higher risk of adverse pregnancy outcomes in women exposed to TPT during pregnancy similar to findings reported by Theron et al 2021 using the same definition of composite adverse pregnancy outcomes that focusses on fetal outcome.

The absence of significant increased risk of adverse pregnancy outcomes may reflect lower baseline risk in our cohort, as all women were already on ART at conception, and achieved viral suppression during pregnancy, factors known to mitigate adverse pregnancy outcomes.

A major strength of this study is its use of routine data from multiple high-burden HIV/TB clinics, enhancing generalizability in similar programmatic settings. Such data allow practical evaluation of intervention safety where randomized trials may not be feasible.

Limitations include potential misclassification or underreporting of early pregnancy losses, preterm births, congenital anomalies, and maternal deaths, due to inadequate routine documentation and limited surveillance systems. Most women initiated antenatal care in the second trimester, reducing capture of early losses. Data on some potential confounders such as syphilis, hepatitis, multiple gestation, nutritional status, smoking, were unavailable. The study was powered to detect at least a 10% absolute difference in the proportion of composite adverse outcomes; however, the observed difference was smaller, and overall event rates were low, which may have limited statistical power to detect modest or rare effects. To mitigate potential sparse data bias, we conducted sensitivity analyses using Firth’s penalized logistic regression, and results remained consistent. Furthermore, although 538 participants were initially sampled from the Uganda EMR, pregnancy records for only 521 women could be identified and linked to paper-based maternity and postnatal registers, resulting in some missing data. Another limitation was the sparse availability of viral load and CD4 measurements in routine care, necessitating a relaxed baseline assessment window that included measurements obtained up to one week after TPT initiation for some women. Lastly, immortal-time bias may have occurred because TPT initiation could occur after the first antenatal visit, requiring exposed women to remain free of adverse pregnancy outcomes until treatment initiation. Although a time-varying exposure analysis was not feasible due to incomplete outcome dates, this bias is unlikely to have materially affected our findings, as a secondary analysis showed no significant differences in outcomes between women initiating TPT in the first trimester and those initiating treatment later in pregnancy.

Despite of the above limitations, the use of linked EMR data across multiple clinics strengthens the generalizability and reliability of our findings in a similar routine programmatic setting.

### Study implication

These findings support continued provision of isoniazid-based TPT for eligible pregnant women living with HIV in high TB/HIV burden settings, without evidence of substantial increased risk. However, non-significant risk signals by trimester underscore the need for strengthened pharmacovigilance, improved documentation, and larger prospective studies to guide optimal timing and monitoring of TPT during pregnancy.

## Conclusion

Our findings suggest a slightly higher, though not statistically significant, risk of adverse pregnancy outcomes among women exposed to six-month isoniazid TPT within routine HIV and antenatal care in Uganda. As TPT scale-up continues in high TB/HIV-burden settings, systematic monitoring through pharmacovigilance and improved documentation of adverse events and maternal risk factors remains essential to safeguard maternal and neonatal health while advancing TB prevention goals.

## Supporting information

S1 Table Variables with missing data and imputation models used. (DOCX)

S2 Table Standardized mean difference (SMD) before and after IPTW weighting. (DOCX)

S1 Fig Love plot showing standardized mean differences for baseline covariates before (unadjusted) and after (IPTW‑adjusted) weighting. Legend: The dashed vertical line indicates the 0.10 threshold for acceptable balance. Covariates are ordered in ascending order according the SMDs before (unadjusted).
(TIF)

S2 Fig Histogram of stabilized inverse probability of treatment weights. (TIF)

S3 Table Adverse pregnancy outcomes by participants’ characteristics*. Legend: *Adverse pregnancy outcomes defined as proportion of composite outcome that includes any of the following: spontaneous abortion/miscarriage, stillbirth, preterm delivery, low birth weight, congenital anomalies, neonatal death, infant death, maternal death or unknown pregnancy outcomes/missing pregnancy outcome (also includes LTFU and transfer-out). Frequencie of individual pregnancy outcomes by TPT exposure groups are presented in Table 2. ‡ P-values derived using Mann-Whitney Wilcoxon rank-sum test to comparing medians across groups, else P-values were derived using Pearson Chi-square test. P-values compare prevalence of adverse pregnancy outcomes between the exposed and non-exposed to TPT during pregnancy † High blood pressure defined as systolic blood pressure >=140 or diastolic blood pressure >=90.
(DOCX)

S4 Table Detailed exposure levels and detailed outcome levels. Legend: *TPT exposure was defined based on the timing of initiation rather than completion, due to incomplete documentation of treatment completion in the paper-based TPT register. Therefore, women who initiated TPT before experiencing an adverse pregnancy outcome were considered exposed. **Includes those who were never TPT at baseline, i.e., pregnancy follow-up start at clinic
(DOCX)

S5 Table Composite pregnancy outcome by 3 TPT status levels. Legend: *TPT exposure was defined based on the timing of initiation rather than completion, due to incomplete documentation of treatment completion in the paper-based TPT register. Therefore, women who initiated TPT before experiencing an adverse pregnancy outcome were considered exposed. † Pearson Chi-square p-value comparing proportion of adverse pregnancy outcomes across the 3 TPT status levels
(DOCX)

S6 Table Weighted odds ratios and 95% confidence intervals for the impact of Isoniazid TPT monotherapy taken during pregnancy on adverse pregnancy outcomes. Estimates from 10, 20 and 30 imputations. Legend: OR = odds ratio; IPTW = inverse probability of treatment weighting; TPT = tuberculosis preventive therapy. ¥ Pooled Odds Ratios and their 95% confidence intervals (95%CI) were estimated from Pooled Weighted Logistic Regression. Propensity scores used to generate Inverse Probability Treatment weights (IPTW) were calculated using a logistic regression model with covariates: healthy facility (study site), maternal age, body mass index, anemia during antenatal care, ART duration, CD4 < 200, viral load <200, ART regimen, calendar year of ART start (pre/post-September 2018), and parity ≥2. Missing data were completed using Multiple Imputation with chained equations using 10 imputations, via predictive mean matching for continuous and logistic model for binary variables. See supporting information Table S1 for details on specific imputation models used.
(DOCX)

S7 Table Participants characteristics by with versus without observed outcomes stratified by TPT exposure during pregnancy. Legend: ‡ P-values derived using Man-Whitney Wilcoxon rank-sum test to comparing medians across groups, else P-values were derived using Pearson Chi-square test. § Fisher’s exact p-values.
(DOCX)

S1 Data Dataset with information analyzed. (XLSX)

S1 File STROBE Checklist — checklist of items that should be included in reports of observational studiesstudies. (DOCX)

## Abbreviations

WHO: World Health Organization
HIV: Human Immunodeficiency Virus
AIDS: Acquired Immune Deficiency Syndrome
TB: Tuberculosis
TPT: Tuberculosis Preventive Treatment
IPT: Isoniazid Preventive Therapy
ART: Antiretroviral Therapy
PLHV: People Living with HIV
WLHIV: Women Living with HIV
Uganda MoH: Uganda Ministry of Health
EMR: Electronic Medical Record
IPTW: Inverse Probability of Treatment Weighting
