# Supplementary material for: Isoniazid monotherapy for tuberculosis prevention during pregnancy and adverse outcomes in women living with HIV in Uganda: A propensity score–weighted analysis of routine care data
Source: PLOS Glob Public Health. 2026 Sep 25;6(9):e0005829. doi: 10.1371/journal.pgph.0005829 (PMC13614580; doi:10.1371/journal.pgph.0005829)
Supplement: Supplementary file 1 [file pgph.0005829.s017.pdf]

"Effect of tuberculosis preventive treatment on adverse pregnancy outcomes in women living with HIV in Uganda: an inverse probability for treatment weighting analysis of routine care data"

Comments:

Title- The title can be informative and does not explicitly mention the observational study design

Adverse outcomes should be specified as adverse pregnancy outcomes for better clarity and explicitly

Introduction- The background gives good context, but the knowledge gap can be simplified and emphasized and prevalence of TB in pregnant woman to be mentioned in the Uganda and specific complications noted in pregnancy due to IPT to be mentioned with prevalence from previous studies.

Methods:

Study setting- Prevalence of HIV and TB in the study setting, maternal outcomes in the given area to be mentioned.

Study population: Explain the expanded TPT provision

Sample size: Statistical software, formula and justification of 10% difference used to be mentioned

Sampling Participation selection to specify the total eligible women before the sample and provide the number selected from each site. Describe in detail the systematic random sampling method.

Variables and outcomes: Clarify the data extraction and specify how the data from different sources were recorded, and describe how discrepancies were resolved. The overlap between low birth weight, stillbirth, and neonatal complications should be clarified. Furthermore, clinically relevant maternal adverse events such as drug-induced liver injury/hepatotoxicity and other serious maternal complications should be considered, or their exclusion should be justified.

Missing data – Justify why LOCF and MICE both approaches were used,

Propensity score- Mention the variables used for the propensity scores

Table: Redefine primary composite outcomes, avoid overlapping outcomes. Instead of a risk difference, a risk ratio would be meaningful with 95% confidence interval. Improve table formatting and align percentages and CI consistency to improve readability.

Discussion:

Mention the bias involved in the study- explain the selection bias for record data and information bias – documentation and under-reporting of programmatic data, impact of missing data.

Strength- The statement on generalizability should be interpreted cautiously, and it is overstated; findings may be generalizable to comparable settings

Study implications and conclusion- inconsistencies and contradictory statements are used. The implication should avoid implying evidence of safety based solely on a non-significant association and instead emphasise that no statistically significant association was observed.
