# Supplementary material for: Isoniazid monotherapy for tuberculosis prevention during pregnancy and adverse outcomes in women living with HIV in Uganda: A propensity score–weighted analysis of routine care data
Source: PLOS Glob Public Health. 2026 Sep 25;6(9):e0005829. doi: 10.1371/journal.pgph.0005829 (PMC13614580; doi:10.1371/journal.pgph.0005829)
Supplement: Supplementary file 2 [file pgph.0005829.s001.docx]

**S1 Table: Variables with missing data and imputation models used**

| **Variable** | **Levels** | **Missing values** | **Imputation model** |
| --- | --- | --- | --- |
| Adverse pregnancy outcome | [0=No, 1=Yes] | 49/521 (9%) | Logistic multiple regression |
| Maternal age | Numeric (in years) | 3/521 (<1%) | Predictive Mean Matching |
| Body Mass Index at pregnancy | Numeric (in Kgs/m^2^) | 38/521 (7%) | Predictive Mean Matching |
| Baseline CD4 count at ART start <200 cells/mL | [0=No, 1=Yes] | 130/521 (25%) | Logistic multiple regression |
| Viral Load at pregnancy <200 copies/mL | [0=No, 1=Yes] | 186/521 (36%) | Logistic multiple regression |
| Parity ≥ 2 | [0=No, 1=Yes] |  | Logistic multiple regression |
