# Supplementary material for: Isoniazid monotherapy for tuberculosis prevention during pregnancy and adverse outcomes in women living with HIV in Uganda: A propensity score–weighted analysis of routine care data
Source: PLOS Glob Public Health. 2026 Sep 25;6(9):e0005829. doi: 10.1371/journal.pgph.0005829 (PMC13614580; doi:10.1371/journal.pgph.0005829)
Supplement: Supplementary file 3 [file pgph.0005829.s002.docx]

**S2 Table: Standardized mean difference (SMD) before and after IPTW weighting**

| **Variable** | **SMD unweighted (Before)** | **SMD weighted (After)** |
| --- | --- | --- |
| Age at pregnancy | -0.09 | 0.034 |
| BMI at pregnancy | 0.117 | 0.001 |
| Haemoglobin <11 g/dL | -0.111 | 0.031 |
| ART duration (years) | -0.254 | 0.025 |
| CD4 at ART start <200 cells/mL | -0.069 | 0.002 |
| Viral Load base <200 copies/mL | 0.113 | 0.006 |
| ART start after September 2018 | 0.202 | -0.016 |
| Parity >2 | 0.046 | 0.014 |
| Health Facility 1 | 0.233 | -0.012 |
| Health Facility 2 | -0.128 | -0.004 |
| Health Facility 3 | -0.119 | 0.033 |
| Health Facility 5 | -0.083 | -0.012 |
| Health Facility 4 | 0.097 | -0.004 |
