# Supplementary material for: Isoniazid monotherapy for tuberculosis prevention during pregnancy and adverse outcomes in women living with HIV in Uganda: A propensity score–weighted analysis of routine care data
Source: PLOS Glob Public Health. 2026 Sep 25;6(9):e0005829. doi: 10.1371/journal.pgph.0005829 (PMC13614580; doi:10.1371/journal.pgph.0005829)
Supplement: Supplementary file 4 [file pgph.0005829.s005.docx]

**S3 Table: Adverse pregnancy outcomes by participants’ characteristics***

| **Characteristics** | **Total** | **Non-adverse pregnancy outcome** | **Adverse pregnancy outcome** | **P-value** |
| --- | --- | --- | --- | --- |
|  | **N=** **472** | **N=** **425 (90.0%)**  **n (%)** | **N=** **47 (10.0%)**  **n (%)** |  |
| Exposed to TPT during pregnancy |  |  |  |  |
| No | 259 | 234 (90.3) | 25 (9.7) | 0.81 |
| Yes | 213 | 191 (89.7) | 22 (10.3) |  |
| Mother’s age in years, median (IQR) | 470 | N=423  28 (24, 31) | N=47  26 (23, 32) | 0.10‡ |
| Parity, n (%) |  |  |  |  |
| < 2 live births | 211 | 186 (88.2) | 25 (11.8) | 0.36 |
| ≥ 2 live births | 218 | 198 (90.8) | 20 (9.2) |  |
| BMI in Kgs/m^2^, median (IQR) | 452 | N=407  24.9 (22.4, 27.6) | N=45  25.4 (22.3, 29.0) | 0.32 ‡ |
| Gestation age at first ANC visit in weeks, median (IQR) | 398 | N=358  24 (18, 30) | N=40  22 (14, 28) | 0.11 ‡ |
| Had >1 ANC visits, n (%) |  |  |  |  |
| No | 235 | 210 (89.4) | 25 (10.6) | 0.77 |
| Yes | 184 | 166 (90.2) | 18 (9.8) |  |
| Nadir CD4 count in cells/mL, median (IQR) † | 365 | N=329  388 (237, 580) | N=36  426 (243, 576) | 0.74 ‡ |
| Nadir CD4 count, n (%) |  |  |  |  |
| ≥200 cells/mL | 295 | 265 (89.8) | 30 (10.2) | 0.69 |
| <200 cells/mL | 70 | 64 (91.4) | 6 (8.6) |  |
| Viral load at pregnancy, n (%) |  |  |  |  |
| ≥ 1000 copies/mL | 24 | 22 ( 91.7) | 2 (8.3) | 0.77 |
| < 1000 copies/mL | 295 | 265 (89.8) | 30 (10.2) |  |
| Calendar year of ART start, n (%) |  |  |  |  |
| Before 2018 | 327 | 296 (90.5) | 31 (9.5) | 0.55 |
| After 2018 | 142 | 126 (88.7) | 16 (11.3) |  |
| ART duration in months, median (IQR) | 468 | N=422  23 (0, 53) | N=46  21 (0, 56) | 0.98 ‡ |
| High blood pressure † |  |  |  |  |
| No | 458 | 413 (90.2) | 45 (9.8) | 0.58 |
| Yes | 14 | 12 (85.7) | 2 (14.3) |  |
| Aneamia (haemoglobin<11g/dl) |  |  |  |  |
| No | 415 | 377 (90.8) | 38 (9.2) | 0.12 |
| Yes | 57 | 48 (84.2) | 9 (15.8) |  |
| ART regimen during pregnancy |  |  |  |  |
| DTG based | 28 | 22 (78.6) | 6 (21.4) | 0.17 |
| EFV based | 416 | 376 (90.4) | 40 (9.6) |  |
| NVP based | 10 | 9 (90.0) | 1 (10.0 |  |
| PI (i.e. ATVr OR LPVr) | 9 | 9 (100.0) | 0 |  |

*Adverse pregnancy outcomes defined as proportion of composite outcome that includes any of the following: spontaneous abortion/miscarriage, stillbirth, preterm delivery, low birth weight, congenital anomalies, neonatal death, infant death, maternal death or unknown pregnancy outcomes/missing pregnancy outcome (also includes LTFU and transfer-out). Frequencie of individual pregnancy outcomes by TPT exposure groups are presented in Table 2.

‡ P-values derived using Mann-Whitney Wilcoxon rank-sum test to comparing medians across groups, else P-values were derived using Pearson Chi-square test. P-values compare prevalence of adverse pregnancy outcomes between the exposed and non-exposed to TPT during pregnancy

† High blood pressure defined as systolic blood pressure >=140 or diastolic blood pressure >=90.
