# Supplementary material for: Isoniazid monotherapy for tuberculosis prevention during pregnancy and adverse outcomes in women living with HIV in Uganda: A propensity score–weighted analysis of routine care data
Source: PLOS Glob Public Health. 2026 Sep 25;6(9):e0005829. doi: 10.1371/journal.pgph.0005829 (PMC13614580; doi:10.1371/journal.pgph.0005829)
Supplement: Supplementary file 5 [file pgph.0005829.s006.docx]

**S4 Table: Detailed exposure levels and detailed outcome levels**

| **Pregnancy outcome** | **TPT exposure*** | | | | | **Total** | **TPT status at baseline** | |
| --- | --- | --- | --- | --- | --- | --- | --- | --- |
|  | **Never**  **N=113**  **n (%)** | **Completed prior**  **N=146**  **n (%)** | **1^st^ Trimester**  **N=130**  **n (%)** | **2^nd^ Trimester**  **N=54**  **n (%)** | **3^rd^ Trimester**  **N=29**  **n (%)** | **N=472**  **n (%)** | **Never on TPT at baseline****  **N=** **326** | **Completed TPT prior**  **N=** **146** |
| **Composite outcome:** adverse pregnancy outcome | **11 (9.7)** | **14 (9.6)** | **12 (9.2)** | **4 (7.4)** | **6 (20.7)** | **44 (9.3)** | **33 (10.1)** | **14 (9.6)** |
| *p-value* | *0.358†* | | | | |  | *0.858* | |
| **Individual outcomes** |  |  |  |  |  |  |  |  |
| Normal live baby | 102 (90.3) | 132 (90.4) | 118 (90.8) | 50 (92.6) | 23 (79.3) | 425 (90.1) | 293 (89.9) | 132 (90.4) |
| Low birth weight | 5 (4.2) | 5 (3.4) | 5 (3.8) | 0 | 1 (3.5) | 16 (3.4) | 11 (3.4) | 5 (3.4) |
| Spontaneous abortion/Miscarriage | 2 (1.8) | 4 (2.8) | 1 (0.8) | 1 (1.9) | 0 | 8 (1.7) | 4 (1.2) | 4 (2.7) |
| Stillbirth | 1 (0.9) | 1 (0.7) | 3 (2.3) | 0 | 4 (13.8) | 9 (1.9) | 8 (2.4) | 1 (0.7) |
| Neonatal death | 0 | 1 (0.7) | 0 | 0 | 0 | 1 (0.2) | 0 | 1 (0.7) |
| Neonatal complication at birth: Breathing difficulty | 2 (1.8) | 2 (1.4) | 3 (2.3) | 3 (5.7) | 1 (3.4) | 11 (2.3) | 9 (2.8) | 2 (1.4) |
| Maternal death | 1 (0.9) | 1 (0.7) | 0 | 0 | 0 | 2 (0.4) | 1 (0.3) | 1 (0.7) |
| *P-value* | *0.050†* | | | | |  | *0.361* | |

*TPT exposure was defined based on the timing of initiation rather than completion, due to incomplete documentation of treatment completion in the paper-based TPT register. Therefore, women who initiated TPT before experiencing an adverse pregnancy outcome were considered exposed.

**Includes those who were never TPT at baseline, i.e. pregnancy follow-up start at clinic

† Denotes Fisher’s exact p-value, else Pearson Chi-square p-value
