# Supplementary material for: Isoniazid monotherapy for tuberculosis prevention during pregnancy and adverse outcomes in women living with HIV in Uganda: A propensity score–weighted analysis of routine care data
Source: PLOS Glob Public Health. 2026 Sep 25;6(9):e0005829. doi: 10.1371/journal.pgph.0005829 (PMC13614580; doi:10.1371/journal.pgph.0005829)
Supplement: Supplementary file 6 [file pgph.0005829.s007.docx]

**Table S5: Composite pregnancy outcome by 3 TPT status levels**

| **TPT status** | **Adverse pregnancy outcome** | | | **p-value†** |
| --- | --- | --- | --- | --- |
|  | **No**  **N=425** | **Yes**  **N=47** | **Total**  **N=472** |  |
| Never exposed to TPT | 102 (90.3) | 11 (9.7) | 113 | 0.970 |
| Pre-pregnancy TPT completion | 132 (90.4) | 14 (9.6) | 146 |  |
| TPT exposure during pregnancy | 191 (89.7) | 22 (10.3) | 213 |  |

*TPT exposure was defined based on the timing of initiation rather than completion, due to incomplete documentation of treatment completion in the paper-based TPT register. Therefore, women who initiated TPT before experiencing an adverse pregnancy outcome were considered exposed.

† Pearson Chi-square p-value comparing proportion of adverse pregnancy outcomes across the 3 TPT status levels
