# Supplementary material for: Isoniazid monotherapy for tuberculosis prevention during pregnancy and adverse outcomes in women living with HIV in Uganda: A propensity score–weighted analysis of routine care data
Source: PLOS Glob Public Health. 2026 Sep 25;6(9):e0005829. doi: 10.1371/journal.pgph.0005829 (PMC13614580; doi:10.1371/journal.pgph.0005829)
Supplement: Supplementary file 7 [file pgph.0005829.s008.docx]

**S6 Table: Weighted odds ratios and 95% confidence intervals for the impact of Isoniazid TPT monotherapy taken during pregnancy on adverse pregnancy outcomes. Estimates from 10, 20 and 30 imputations**

|  | Odds Ratio (95% CI) | *P*-value |
| --- | --- | --- |
|  |  |  |
| IPTW weighted Logistic regression model (10 imputations) | 1.06 (0.68, 1.63) | 0.81 |
| IPTW weighted Logistic regression model (20 imputations) | 1.05 (0.58, 1.88) | 0.81 |
| IPTW weighted Logistic regression model (30 imputations) | 1.04 (0.58, 1.87) | 0.90 |

OR = odds ratio; IPTW = inverse probability of treatment weighting; TPT = tuberculosis preventive therapy.

¥ Pooled Odds Ratios and their 95% confidence intervals (95%CI) were estimated from Pooled Weighted Logistic Regression. Propensity scores used to generate Inverse Probability Treatment weights (IPTW) were calculated using a logistic regression model with covariates: healthy facility (study site), maternal age, body mass index, anemia during antenatal care, ART duration, CD4 <200, viral load <200, ART regimen, calendar year of ART start (pre/post-September 2018), and parity ≥2. Missing data were completed using Multiple Imputation with chained equations using 10 imputations, via predictive mean matching for continuous and logistic model for binary variables. See supporting information Table S1 for details on specific imputation models used.
