# Supplementary material for: Isoniazid monotherapy for tuberculosis prevention during pregnancy and adverse outcomes in women living with HIV in Uganda: A propensity score–weighted analysis of routine care data
Source: PLOS Glob Public Health. 2026 Sep 25;6(9):e0005829. doi: 10.1371/journal.pgph.0005829 (PMC13614580; doi:10.1371/journal.pgph.0005829)
Supplement: Supplementary file 8 [file pgph.0005829.s009.docx]

**S7 Table: Participants characteristics by with versus without observed outcomes stratified by TPT exposure during pregnancy**

| characteristics | Exposed to TPT during pregnancy | | | Not exposed to TPT during pregnancy | | |
| --- | --- | --- | --- | --- | --- | --- |
|  | Outcome available  n/N (%) | Outcome unavailable  n/N (%) | p-value | Outcome available  n/N (%) | Outcome unavailable  n/N (%) | p-value |
|  |  |  |  |  |  |  |
| Age in years, median (IQR) | 28 (24, 31) | 28 (24, 32) | 0.992‡ | 28 (25, 32) | 26 (23, 30) | 0.058‡ |
| Parity ≥ 2 births | 107/ 202 (53.0) | 6/13 (46.2) | 0.633 | 111/227(48.9) | 14/31 (45.2) | 0.696 |
| BMI in Kgs/m^2^, median (IQR) | 24.9 (22.5, 27.5) | 25.3 (23.1, 28.4) | 0.619‡ | 25.0 (22.2, 27.8) | 23.9 (20.5, 27.7) | 0.542‡ |
| CD4 count <200 cells/mL | 27/167 (16.2) | 1/12 (8.3) | 0.694§ | 43/198 (21.7) | 2/14 (14.3) | 0.739§ |
| Viral load < 200 copies/mL | 130/141 (92.2) | 6/9 (66.7) | 0.039 | 157/178 (88.2) | 6/7 (85.7) | 0.594 |
| ART start after 2018 | 90/213 (42.3) | 6/14 (42.9) | 0.999 | 52/256 (20.3) | 5/19 (26.3) | 0.559 |
| ART regimen at pregnancy |  |  |  |  |  |  |
| DTG based | 11/209 (5.3) | 1/14 (7.1) | 0.724§ | 17/254 (6.7) | 4/19 (21.0) | 0.150§ |
| EFV based | 191/209 (91.4) | 13/14 (92.9) |  | 225/254 (88.6) | 15/19 (79.0) |  |
| NVP based | 3/209 (1.4) | 0 |  | 7/254 (2.7) | 0 |  |
| ATV/r or LPV/r | 4/209 (1.9) | 0 |  | 5/254 (2.0) | 0 |  |

‡ P-values derived using Man-Whitney Wilcoxon rank-sum test to comparing medians across groups, else P-values were derived using Pearson Chi-square test. § Fisher’s exact p-values.
