# Supplementary material for: Isoniazid monotherapy for tuberculosis prevention during pregnancy and adverse outcomes in women living with HIV in Uganda: A propensity score–weighted analysis of routine care data
Source: PLOS Glob Public Health. 2026 Sep 25;6(9):e0005829. doi: 10.1371/journal.pgph.0005829 (PMC13614580; doi:10.1371/journal.pgph.0005829)
Supplement: Supplementary file 9 [file pgph.0005829.s011.docx]

S1 STROBE Checklist — checklist of items that should be included in reports of observational studies

|  | Item No. | Recommendation | Page  No. | Relevant text from manuscript |
| --- | --- | --- | --- | --- |
| **Title and abstract** | 1 | (*a*) Indicate the study’s design with a commonly used term in the title or the abstract | 1 | … an inverse probability for treatment weighting analysis of routine care data |
|  |  | (*b*) Provide in the abstract an informative and balanced summary of what was done and what was found | 2 | Using routinely collected data from five urban HIV clinics in Kampala, Uganda, we conducted a retrospective cohort study of 521 pregnant women living with HIV on ART between 2016 and 2022 to assess whether six‑month isoniazid preventive therapy (IPT) during pregnancy was associated with adverse pregnancy outcomes. Applying inverse probability of treatment weighting to adjust for confounding, we found no statistically significant differences in composite or individual adverse pregnancy outcomes between women exposed and unexposed to IPT, with findings remaining robust across sensitivity and subgroup analyses. |
| Introduction | | | |  |
| Background/rationale | 2 | Explain the scientific background and rationale for the investigation being reported | 4 | Although World Health Organization (WHO) recommends Tuberculosis preventive treatment (TPT) for pregnant women living with HIV, concerns about isoniazid safety during pregnancy persist due to mixed evidence from clinical trials and observational studies. In Uganda, a high TB/HIV burden setting with widespread TPT implementation, real-world data on pregnancy safety outcomes are limited, providing the rationale for this investigation. |
| Objectives | 3 | State specific objectives, including any prespecified hypotheses | 4 | This study aimed to assess the effect of six‑month isoniazid preventive therapy during pregnancy on adverse pregnancy outcomes among women living with HIV in Uganda, including miscarriage, stillbirth, low birth weight, preterm delivery, congenital anomalies, and maternal or neonatal death, using routinely collected programmatic data. |
| Methods | | | |  |
| Study design | 4 | Present key elements of study design early in the paper | 4-5 | We conducted a retrospective cohort study using routinely collected HIV and antenatal care data from five public PEPFAR-supported primary health facilities in Kampala, Uganda. |
| Setting | 5 | Describe the setting, locations, and relevant dates, including periods of recruitment, exposure, follow-up, and data collection | 4-5 | The study was conducted at five public HIV clinics in Kampala, Uganda. Pregnant women living with HIV on ART between January 2016 and December 2022 were included, with IPT exposure occurring during pregnancy and follow‑up through documented pregnancy outcomes. Data were obtained from routine clinical registers and electronic medical records and extracted between July 2020 and December 2023. |
| Participants | 6 | (*a*) *Cohort study*—Give the eligibility criteria, and the sources and methods of selection of participants. Describe methods of follow-up  *Case-control study*—Give the eligibility criteria, and the sources and methods of case ascertainment and control selection. Give the rationale for the choice of cases and controls  *Cross-sectional study*—Give the eligibility criteria, and the sources and methods of selection of participants | 4-5 | Pregnant women living with HIV aged ≥15 years on ART between 2016 and 2022 were retrospectively identified from five public clinics in Kampala using EMR data. Women with unknown ART status, prolonged gaps in care, or TPT initiation before ART were excluded. Participants were selected using systematic random sampling stratified by TPT exposure. Follow‑up extended from pregnancy documentation through recorded pregnancy and early postpartum outcomes using linked routine clinical records.. |
|  |  | (*b*) *Cohort study*—For matched studies, give matching criteria and number of exposed and unexposed  *Case-control study*—For matched studies, give matching criteria and the number of controls per case |  | Not applicable |
| Variables | 7 | Clearly define all outcomes, exposures, predictors, potential confounders, and effect modifiers. Give diagnostic criteria, if applicable |  | Adverse pregnancy outcomes were defined using composite and individual measures including miscarriage, stillbirth, low birth weight, congenital anomalies, and maternal or neonatal death. Exposure was six‑month isoniazid TPT during pregnancy, classified overall and by trimester. Covariates included maternal demographic, reproductive, and HIV‑related clinical factors, with viral load, baseline CD4 count, and timing of IPT initiation assessed as potential effect modifiers. |
| Data sources/ measurement | 8* | For each variable of interest, give sources of data and details of methods of assessment (measurement). Describe comparability of assessment methods if there is more than one group | 7-8 | Study variables were derived from routine paper-based registers and the national EMR used in HIV, antenatal, maternity, and postnatal services. IPT exposure, pregnancy outcomes, and covariates were assessed using standardized clinical documentation, with identical data sources and assessment methods applied to exposed and unexposed groups. |
| Bias | 9 | Describe any efforts to address potential sources of bias | 8, 15-16 | Bias was addressed through use of IPTW to control for measured confounding, multiple imputation for missing data, sensitivity analyses including penalized regression to assess sparse data bias, and consistent outcome ascertainment across exposure groups. Residual confounding from unmeasured maternal factors remained possible. |
| Study size | 10 | Explain how the study size was arrived at | 5 | The sample size was calculated to detect a ≥10% absolute difference in adverse pregnancy outcomes with 80% power at α=0.05, assuming a 17% baseline risk. Although 538 women were planned, 521 were included due to missing records. |

Continued on next page

| Quantitative variables | 11 | Explain how quantitative variables were handled in the analyses. If applicable, describe which groupings were chosen and why | 8 | Continuous variables were analyzed using appropriate summary statistics and modeled continuously in propensity scores, with predefined categorical groupings applied for clinically relevant subgroup analyses. Missing quantitative data were addressed using last observation carried forward for longitudinal measures and multiple imputation for other variables. |
| --- | --- | --- | --- | --- |
| Statistical methods | 12 | (*a*) Describe all statistical methods, including those used to control for confounding | 8 | Statistical analyses included descriptive comparisons, IPTW using propensity scores to control for confounding, weighted logistic regression to estimate effects, multiple imputation for missing data, and sensitivity analyses including penalized regression and subgroup analyses. |
|  |  | (*b*) Describe any methods used to examine subgroups and interactions | 8 | Subgroup analyses were performed using stratified weighted logistic regression to assess potential effect modification by CD4 count, viral load, age, BMI, timing of IPT initiation, and ART initiation year relative to dolutegravir rollout. |
|  |  | (*c*) Explain how missing data were addressed | 8 | Missing covariate data were handled using multiple imputation by chained equations, while longitudinal weight data used last observation carried forward. Sensitivity analyses were performed to assess robustness to missingness. |
|  |  | (*d*) *Cohort study*—If applicable, explain how loss to follow-up was addressed  *Case-control study*—If applicable, explain how matching of cases and controls was addressed  *Cross-sectional study*—If applicable, describe analytical methods taking account of sampling strategy | 8 | Pregnancy outcomes were retrospectively ascertained by linking HIV care, antenatal, maternity, and postnatal records. Women with completely missing outcome data due to loss to follow‑up were excluded, and outcome analyses were conducted using complete cases. |
|  |  | (*e*) Describe any sensitivity analyses | 8 | Sensitivity analyses included unadjusted, covariate‑adjusted, and Firth’s penalized logistic regression models to assess robustness to model specification and sparse outcome bias. |
| Results | | | | |
| Participants | 13* | (a) Report numbers of individuals at each stage of study—eg numbers potentially eligible, examined for eligibility, confirmed eligible, included in the study, completing follow-up, and analysed | 9 | Of 13,401 pregnant women living with HIV recorded at the study facilities, 538 were sampled and 521 had linked pregnancy outcome records and were included in the final analysis (227 IPT‑exposed and 294 unexposed). |
|  |  | (b) Give reasons for non-participation at each stage | 9 | Non‑participation occurred due to sampling requirements and missing or unlinked paper‑based pregnancy records. Of 538 sampled women, 17 were excluded because pregnancy outcomes could not be ascertained, and 521 were included in the final analysis. No participants declined participation, as the study used retrospective routine data. |
|  |  | (c) Consider use of a flow diagram |  | Not used flow diagram since it had few steps. Just described flow of numbers |
| Descriptive data | 14* | (a) Give characteristics of study participants (eg demographic, clinical, social) and information on exposures and potential confounders | 9,10 | Provided |
|  |  | (b) Indicate number of participants with missing data for each variable of interest | 10 | Provided |
|  |  | (c) *Cohort study*—Summarise follow-up time (eg, average and total amount) |  | Not applicable since this was not survival analysis data |
| Outcome data | 15* | *Cohort study*—Report numbers of outcome events or summary measures over time | 11,12 | Provided |
|  |  | *Case-control study—*Report numbers in each exposure category, or summary measures of exposure |  | Not applicable |
|  |  | *Cross-sectional study—*Report numbers of outcome events or summary measures |  | Not applicable |
| Main results | 16 | (*a*) Give unadjusted estimates and, if applicable, confounder-adjusted estimates and their precision (eg, 95% confidence interval). Make clear which confounders were adjusted for and why they were included | 13 | Provided |
|  |  | (*b*) Report category boundaries when continuous variables were categorized | 10, 12 | Provided, i.e. CD4 count, maternal viral load, birth weight |
|  |  | (*c*) If relevant, consider translating estimates of relative risk into absolute risk for a meaningful time period | 12 | Translated where applicable (Table 2) |

Continued on next page

| Other analyses | 17 | Report other analyses done—eg analyses of subgroups and interactions, and sensitivity analyses | 13, 14 | Provided |
| --- | --- | --- | --- | --- |
| Discussion | | | | |
| Key results | 18 | Summarise key results with reference to study objectives | 15 | Provided |
| Limitations | 19 | Discuss limitations of the study, taking into account sources of potential bias or imprecision. Discuss both direction and magnitude of any potential bias | 16, 17 | Provided |
| Interpretation | 20 | Give a cautious overall interpretation of results considering objectives, limitations, multiplicity of analyses, results from similar studies, and other relevant evidence | 17 | Provided |
| Generalisability | 21 | Discuss the generalisability (external validity) of the study results | 16, 17 | Provided |
| Other information | |  | | |
| Funding | 22 | Give the source of funding and the role of the funders for the present study and, if applicable, for the original study on which the present article is based | 18 | Provided |

*Give information separately for cases and controls in case-control studies and, if applicable, for exposed and unexposed groups in cohort and cross-sectional studies.

**Note:** An Explanation and Elaboration article discusses each checklist item and gives methodological background and published examples of transparent reporting. The STROBE checklist is best used in conjunction with this article (freely available on the Web sites of PLoS Medicine at http://www.plosmedicine.org/, Annals of Internal Medicine at http://www.annals.org/, and Epidemiology at http://www.epidem.com/). Information on the STROBE Initiative is available at www.strobe-statement.org.
