# Supplementary material for: Isoniazid monotherapy for tuberculosis prevention during pregnancy and adverse outcomes in women living with HIV in Uganda: A propensity score–weighted analysis of routine care data
Source: PLOS Glob Public Health. 2026 Sep 25;6(9):e0005829. doi: 10.1371/journal.pgph.0005829 (PMC13614580; doi:10.1371/journal.pgph.0005829)
Supplement: Supplementary file 10 [file pgph.0005829.s012.docx]

**Review report**

**L1, L96**: Title, objective: be consistent with the words you use: Impact??? Association? The use of “**estimated effect**” could be scientifically sound.

**Abstract**: Make a structured abstract

**L63-L70:** Very long sentences. Don’t exceed 25 for one sentence. Check the whole study.

**L128-145**: Relocate these paragraphs in introduction as they explain the program. Instead, it mentions what was based on from this program to select the study participants or data to include in this study.

**L153**: **A total sample size of 538 participants**. How have you found this sample size. By which method or sample size formula have you used to get this sample size. Could you mention the study population number. This means all the population that were found the 5 selected health facilities in Kampala.

**L238-L243:**

- Relocate this. This should be written after the population study before sampling procedure. Because it helps to understand the flow and digestibility of information. Eg. Study design- sample-selection criteria and so on.
- Make sure to reference the baseline risks for ensuring that they are evidenced-based not arbitrary. Because 17% baseline risk in the unexposed group will influence the results and the conclusion.
- Explain in few words in sample size. How you have assigned the exposed and non-exposed sample.
- There is a lack of summarized protocol of information flow from study population number- exclusion number -total sample number and group sample number.
- Mention the software that you have used.

**382-Discussion**

- Discuss the use of small sample size with more missing data and non-compliance of IPT through out the 3 trimesters.
- Discuss this study implications.
