# Supplementary material for: Isoniazid monotherapy for tuberculosis prevention during pregnancy and adverse outcomes in women living with HIV in Uganda: A propensity score–weighted analysis of routine care data
Source: PLOS Glob Public Health. 2026 Sep 25;6(9):e0005829. doi: 10.1371/journal.pgph.0005829 (PMC13614580; doi:10.1371/journal.pgph.0005829)
Supplement: Supplementary file 11 [file pgph.0005829.s014.docx]

The Editor,

PLOS Global Public Health

Date: 29^th^ December 2025

Dear Sir,

**RE: RESPONSE TO REVIEWERS’ COMMENTS MANUSCRIPT ID: PGPH-D-25-03149 “Impact of tuberculosis preventive treatment on adverse pregnancy outcomes in women living with HIV in Uganda: an inverse probability for treatment weighting analysis of routine care data”.**

*The authors of the reviewed manuscript thank the academic editor and reviewers for their consideration and careful review of the manuscript. Below, we provide detailed point-by-point responses to the reviewers’ comments in maroon font and italics. We have also tracked the changes in the revised manuscript.*

*In this document, we have also provided page and line numbers as referenced from the tracked manuscript to enable reviewers easily track the changes made as per their comments.*

**5. Review Comments to the Author**

**L1, L96**: Title, objective: be consistent with the words you use: Impact??? Association? The use of “**estimated effect**” could be scientifically sound.

***Response****: Thank you for the comment.*

*We have replaced the term “impact” with “effect” throughout the manuscript.*

**Abstract**: Make a structured abstract

***Response****: Thank you.*

*We have made a structure abstract as guided, showing: Background, Methods, Results and Conclusion (page 2, lines 30 – 61).*

**L63-L70:** Very long sentences. Don’t exceed 25 for one sentence. Check the whole study.

***Response****: Thank you.*

*We have split most of long sentences to reasonable length.*

**L128-145**: Relocate these paragraphs in introduction as they explain the program. Instead, it mentions what was based on from this program to select the study participants or data to include in this study.

***Response****: Thank you.*

*The paragraph on TPT implementation has been transferred from methods section to introduction section, as guided (page 4, lines 89 – 98).*

**L153**: **A total sample size of 538 participants**. How have you found this sample size. By which method or sample size formula have you used to get this sample size. Could you mention the study population number. This means all the population that were found the 5 selected health facilities in Kampala.

***Response****: Thank you.*

*Sample size statement with assumptions was provided (**page 5, lines 135 – 140).*

*The statement reads as follows:*

*“Sample size and statistical power*

*We estimated a sample size of 538 pregnant WLHIV (269 per group) to achieve 80% power to detect a ≥10 percentage-point difference in adverse pregnancy outcomes between TPT-exposed and unexposed groups, assuming a 17% baseline risk (4), with a two-sided alpha of 0.05 using Pearson’s Chi-square test. However, the final sample size was 521 women due to missing patient files and registers at some study facilities.”*

**L238-L243:**

- Relocate this. This should be written after the population study before sampling procedure. Because it helps to understand the flow and digestibility of information. Eg. Study design- sample-selection criteria and so on.

***Response****:*

*The paragraph on sample size and statistical power has been transferred, and has been placed after study population description, and before participants’ selection procedures (page 5, lines 135 – 140).*

- Make sure to reference the baseline risks for ensuring that they are evidenced-based not arbitrary. Because 17% baseline risk in the unexposed group will influence the results and the conclusion.

***Response****: Thank you for the comment*

*Reference for the baseline risks has been inserted (page 5, lines 135 – 140).*

- Explain in few words in sample size. How you have assigned the exposed and non-exposed sample.

***Response****:*

*Assignment to the exposed versus non-exposed was through stratification, as described in the participants’ selection and sampling strategy section. Below we provide the statement in the manuscript (page 5, lines 142 – 147).*

*“Participants’ selection and sampling strategy*

*Using the Uganda EMR system, we generated a sampling frame of eligible WLHIV with documented pregnancy. Participants were proportionally allocated across the five sites and selected using systematic random sampling, stratified by TPT-exposed. The sampling interval was calculated by dividing the total number of eligible women (sampling frame) at each site by the number of participants allocated to that site.”.*

- There is a lack of summarized protocol of information flow from study population number- exclusion number -total sample number and group sample number.

***Response****:*

*The flow of participants has been included in first paragraph of Results (pages 8, lines 252 – 255).*

*The statement reads as below.*

*“Between 2016 and 2022, a total of 13,401 pregnant women living with HIV were recorded in HIV care clinics across the five public primary health care facilities. From this population, 538 women were sampled. Of these, 521 were identified through maternity and postnatal paper-based registers and were included in the final analysis (Table 1).”.*

- Mention the software that you have used.

***Response****:*

*Software used has be mentioned (page 8, lines 247 – 248).*

*The statement reads as below.*

*“Data were cleaned using STATA v.17.0 (Stata Corp, College Station, TX, USA), and analysis performed using R software v.4.3.3 (R Computing, Vienna, Austria).”*

**382-Discussion**

- Discuss the use of small sample size with more missing data and non-compliance of IPT through out the 3 trimesters.

***Response****: Thank you for this comment.*

*We have expanded the Discussion to address the limitations related to the sample size, and missing data. (page 16, lines 418–426).*

*The revised text in the Discussion reads as follows:*

*“The study was powered to detect at least a 10% absolute difference in the proportion of composite adverse outcomes; however, the observed difference was smaller, and overall event rates were low, which may have limited statistical power to detect modest or rare effects. To mitigate potential sparse data bias, we conducted sensitivity analyses using Firth’s penalized logistic regression, and results remained consistent. Furthermore, although 538 participants were initially sampled from the Uganda EMR, pregnancy records for only 521 women could be identified and linked to paper-based maternity and postnatal registers, resulting in some missing data.”*

About “non-compliance of IPT throughout the 3 trimesters”, we defined exposure basing on timing of initiation (i.e. trimester 1, 2 or 3) rather than assessing completion of full IPT course or maintained consistent use across trimesters. (page 7, lines 202 – 206)

*The statement reads as below.*

“*TPT exposure was defined based on the timing of initiation rather than completion, due to incomplete documentation of treatment completion in the paper-based TPT register. Therefore, women who initiated TPT before experiencing an adverse pregnancy outcome were considered exposed.”*

- Discuss this study implications.

***Response****:*

*Study implication has been added (pages 17, lines 429 – 434).*

*The added statement in the Discussion reads as follows:*

*“These findings support continued provision of isoniazid-based TPT for eligible pregnant women living with HIV in high TB/HIV burden settings, without evidence of substantial increased risk. However, non-significant risk signals by trimester underscore the need for strengthened pharmacovigilance, improved documentation, and larger prospective studies to guide optimal timing and monitoring of TPT during pregnancy.”*
