# Supplementary material for: Isoniazid monotherapy for tuberculosis prevention during pregnancy and adverse outcomes in women living with HIV in Uganda: A propensity score–weighted analysis of routine care data
Source: PLOS Glob Public Health. 2026 Sep 25;6(9):e0005829. doi: 10.1371/journal.pgph.0005829 (PMC13614580; doi:10.1371/journal.pgph.0005829)
Supplement: Supplementary file 12 [file pgph.0005829.s015.docx]

The Editor,

PLOS Global Public Health

Date: 2^nd^ May 2026

Dear Sir/Madam,

**RE: RESPONSE TO REVIEWERS’ COMMENTS MANUSCRIPT: PGPH-D-25-03149R1 “Effect of tuberculosis preventive treatment on adverse pregnancy outcomes in women living with HIV in Uganda: an inverse probability for treatment weighting analysis of routine care data”.**

*The authors of the reviewed manuscript thank the academic editor and reviewers for their consideration and careful review of the manuscript. Below, we provide detailed point-by-point responses to the reviewers’ comments in maroon font and italics. We have also tracked the changes in the revised manuscript.*

*In this document, we have also provided page and line numbers as referenced from the tracked manuscript to enable reviewers easily track the changes made as per their comments.*

**Review Comments to the Author**

**Reviewer #2:** Thank you for the revision.

- While IPTW with multiple imputation is appropriate, the manuscript still does not present balance diagnostics to demonstrate that weighting adequately controlled confounding. Please report standardized mean differences before and after weighting and describe the distribution of stabilized weights (or acknowledge this as a limitation if unavailable).

***Response****: Thank you for the comment.*

*We have reported standardized mean differences before and after weighting, and also described the distribution of stabilized weights. In the revised manuscript with track changes, refer to Methods section page 8, lines 245-250, and in results section, page 12, lines 314-319.*

*Statement methods section reads as below:*

*“Covariate balance between TPT‑exposed and unexposed women was assessed using standardized mean differences (SMDs). Balance was evaluated before and after inverse probability of treatment weighting, with SMDs <0.10 considered indicative of adequate balance. Stabilized IPTW weights were used. The distribution of stabilized weights was examined using summary statistics and graphical diagnostics to assess the presence of extreme weights.”*

*Statement results section reads as below:*

“*Standardized mean differences indicated notable covariate imbalance before weighting. After stabilized IPTW, all covariates achieved acceptable balance (absolute SMD < 0.10), demonstrating effective mitigation of measured confounding (Table S2 and Fig S1). The stabilized IPTW weights shows that most weights clustered around 1, with a modest right skewed tail (Fig S2). The absence of extreme weight values suggests adequate covariate overlap and stable estimation of the weighted treatment effect.”*

*A Love plot showing standardized mean differences (SMDs) for baseline covariates before (unadjusted) and after (IPTW adjusted) weighting, has been included in the supplementary file, i.e. Fig S1.*

**Fig S1:** Love plot showing standardized mean differences for baseline covariates before (unadjusted) and after (IPTW‑adjusted) weighting. The dashed vertical line indicates the 0.10 threshold for acceptable balance. Covariates are ordered in ascending order according the SMDs before (unadjusted)

- In addition, as this is an observational study, please state that reporting follows STROBE guidelines and include the completed STROBE checklist in the supplementary material.

***Response****: Thank you.*

*The statement that* *reporting follows STROBE guidelines has been included in statistical methods section. In the revised manuscript with track changes, refer to page 9, lines 262-263.*

*Also, a completed STROBE checklist has been provided in the supplementary material.*

- Finally, please review the manuscript carefully for minor grammatical and wording errors before final submission.

***Response****: Thank you.*

*We have made a careful review of the manuscript and corrected the grammatical and wording errors.*
