# Supplementary material for: Isoniazid monotherapy for tuberculosis prevention during pregnancy and adverse outcomes in women living with HIV in Uganda: A propensity score–weighted analysis of routine care data
Source: PLOS Glob Public Health. 2026 Sep 25;6(9):e0005829. doi: 10.1371/journal.pgph.0005829 (PMC13614580; doi:10.1371/journal.pgph.0005829)
Supplement: Supplementary file 13 [file pgph.0005829.s016.docx]

**Recommendation: Major revision**

The study addresses an important evidence gap and uses valuable routine-care data. However, several concerns regarding exposure definition, missing outcomes, confounding and interpretation require clarification and reanalysis.

**Review comments**

1. Lines 42–49: The missingness of pregnancy outcome data for 49 women is mentioned in the abstract. Clarification on whether multiple imputation was applied only to covariates or also to the outcome.
2. Lines 171–182: Preterm delivery has been removed from the revised abstract and primary composite, but it remains in parts of the objective and the footnote explanation of Tables 2 and 3. Since preterm birth was not reliably documented, it should be removed from all analytical outcome definitions. Consider using “none were documented” rather than “no cases occurred.”
3. Lines 178–218: The exposure definition may introduce immortal-time bias. Follow-up begins at the first documented pregnancy visit, but women are classified as exposed if they initiate IPT later during pregnancy. Women must therefore remain pregnant and event-free until IPT initiation to enter the exposed group, while earlier events are assigned to the comparison group. This is not an intention-to-treat analysis. Please consider a time-varying exposure analysis or a prespecified gestational landmark that aligns eligibility, treatment assignment and follow-up. If reanalysis is not feasible, this limitation should be acknowledged and causal interpretations avoided.
4. Lines 209–218 and 273–277: The comparison group should not be described as “TPT-unexposed,” because 149 of 294 women had completed IPT before pregnancy. Can be reframed as “IPT initiation during pregnancy versus no initiation during pregnancy.” A sensitivity analysis restricted to women who had never received IPT, or a three-category analysis separating pregnancy initiation, pre-pregnancy completion and never-exposed women, would be meaningful.
5. Lines 220–233: Ensure that all propensity-score covariates were measured before IPT initiation. Viral load was allowed to be measured up to six months after the pregnancy event date and may therefore be post-exposure or post-outcome. The timing of BMI, anaemia and ART regimen should also be clarified. Post-exposure variables should not be treated as baseline confounders.
6. Lines 235–266: The multiple-imputation and IPTW procedures require detailed reporting. Specify whether propensity scores were estimated separately within each imputed dataset, whether the outcome was imputed, how weighted estimates and standard errors were pooled, and the effective sample size after weighting. The adequacy of 10 imputations should be justified, given 25% missing CD4 and 36% missing viral-load data, and also explain the rationale for using the last observation carried forward for weight.
7. Lines 304–327: Pregnancy outcomes were missing for 6.2% of women initiating IPT during pregnancy and 11.9% of the comparison group. A comparison of baseline characteristics among participants with and without observed outcomes by exposure group would provide a better understanding.
8. Lines 349–364: For sparse individual outcomes and zero cells, Fisher’s exact test would be more appropriate than Pearson’s chi-square test.
