# Supplementary material for: Isoniazid monotherapy for tuberculosis prevention during pregnancy and adverse outcomes in women living with HIV in Uganda: A propensity score–weighted analysis of routine care data
Source: PLOS Glob Public Health. 2026 Sep 25;6(9):e0005829. doi: 10.1371/journal.pgph.0005829 (PMC13614580; doi:10.1371/journal.pgph.0005829)
Supplement: Supplementary file 14 [file pgph.0005829.s018.docx]

The Editor,

PLOS Global Public Health

Date: 18^th^ May 2026

Dear Sir/Madam,

**RE: RESPONSE TO REVIEWERS’ COMMENTS MANUSCRIPT: PGPH-D-25-03149R2 “Effect of tuberculosis preventive treatment on adverse pregnancy outcomes in women living with HIV in Uganda: an inverse probability for treatment weighting analysis of routine care data”.**

*The authors of the reviewed manuscript thank the academic editor and reviewers for their consideration and careful review of the manuscript. Below, we provide detailed point-by-point responses to the reviewers’ comments in maroon font and italics. We have also tracked the changes in the revised manuscript.*

*In this document, we have also provided page and line numbers as referenced from the tracked manuscript to enable reviewers easily track the changes made as per their comments.*

**Review Comments to the Author**

**Reviewer #2:** The manuscript has improved substantially and the methodology is presented more clearly in this revised version. The use of IPTW, sensitivity analyses, and acknowledgement of limitations strengthen the paper. I only have a few minor comments remaining before publication consideration.

- The manuscript still occasionally uses causal language such as “effect,” “impact,” “risk,” and “causal inference models” despite the observational retrospective design. Consider replacing these with more cautious terms such as “association,” “odds,” or “propensity score weighted analysis.”

***Response****: Thank you for the comment.*

*We have changed the language throughout the manuscript to avoid causal statements. Also edited the title accordingly. The term “risk” was maintained in some interpretation because this was a cohort study, although it involved retrospective data collection. Therefore, the proportion of cases can be defined as risk.*

*The edited manuscript title read as below:*

*“Isoniazid monotherapy for tuberculosis prevention during pregnancy and adverse outcomes in women living with HIV in Uganda: A Propensity Score–Weighted Analysis of Routine Care Data.”*

- There remains a reporting inconsistency regarding preterm delivery. The abstract and Table 2 include preterm delivery in the composite outcome definition, whereas the Methods section states that preterm birth was excluded due to lack of documentation. This should be harmonized throughout the manuscript.

***Response****: Thank you for highlighting this inconsistency.*

*Preterm delivery has now been removed from the composite outcome definition throughout the manuscript to ensure consistency. Its inclusion in earlier drafts was an oversight, and all relevant sections (including the abstract and Table 2) have been updated accordingly. For completeness, preterm delivery was left in the study aim, however, we reported that there were no document cases in the data.*

- In Table 1, viral load percentages appear to be calculated among participants with available viral load measurements rather than the full cohort. Since 36% of viral load data were missing, the denominators should be clarified in the table footnote to avoid misinterpretation.

***Response****: Thank you for this important comment.*

*We have revised the Table 1 footnote to clarify that summary statistics (medians and percentages) were calculated using available data for each respective variable with missing values. In the revised manuscript with track changes, refer to page 11, lines 287-288.*

*Statement added reads as below:*

*“Summary statistics (medians [IQR] and percentages) were calculated using available-case denominators (i.e., participants with non-missing data for each specific variable).”*

- The category “Other anomalies: Difficulty in breathing” in Table 2 would benefit from clarification, as difficulty in breathing may represent a neonatal complication rather than a congenital anomaly.

***Response****: Thank you for the comment.*

*We agree that the classification of “difficulty in breathing” could be misinterpreted as a neonatal complication rather than a congenital anomaly. In our source data, this variable captured newborns who did not initiate spontaneous breathing at birth and required stimulation and/or resuscitation (e.g., bag-and-mask ventilation).*

*Given this, we have reclassified “difficulty in breathing” as a neonatal complication at birth rather than a congenital anomaly. We also acknowledge that more detailed clinical information was not available, as the data were retrospectively extracted from routinely collected program records.*

*To improve clarity, we have updated the Table 2 categorization and added a detailed explanation in the table footnote. These revisions can be found on pages 13–14, lines 354–361 in the tracked manuscript.*

*Added footnote reads as below:*

*“Difficulty in breathing includes newborn who did not initiate spontaneous breathing at birth and required stimulation and/or resuscitation (e.g., bag-and-mask ventilation).”*

- The subgroup and trimester analyses are interesting, but the very small number of events should be emphasized more clearly when interpreting these findings, particularly regarding stillbirths.

***Response****: Thank you for the comment.*

*The statement on subgroup and trimester has been revised to reflect the small numbers, and avoid over-interpretation. In the revised manuscript with track changes, refer to page 13, lines 337-338.*

*The result on still birth reads as follows:*

*“Although the numbers were small, stillbirth was the most frequently observed adverse event among those who initiated TPT in the third trimester (4/29, 13.8%)”*
