# Supplementary material for: Isoniazid monotherapy for tuberculosis prevention during pregnancy and adverse outcomes in women living with HIV in Uganda: A propensity score–weighted analysis of routine care data
Source: PLOS Glob Public Health. 2026 Sep 25;6(9):e0005829. doi: 10.1371/journal.pgph.0005829 (PMC13614580; doi:10.1371/journal.pgph.0005829)
Supplement: Supplementary file 15 [file pgph.0005829.s019.docx]

**The Editor,**

**PLOS Global Public Health**

**Date: 10th August 2026**

Dear Sir/Madam,

**RE: RESPONSE TO REVIEWERS’ COMMENTS MANUSCRIPT: PGPH-D-25-03149R3 “Effect of tuberculosis preventive treatment on adverse pregnancy outcomes in women living with HIV in Uganda: an inverse probability for treatment weighting analysis of routine care data”.**

The authors of the reviewed manuscript thank the academic editor and reviewers for their consideration and careful review of the manuscript. Below, we provide detailed point-by-point responses to the reviewers’ comments in blue font and *italics*. We have also tracked the changes in the revised manuscript.

In this document, we have also provided page and line numbers as referenced from the tracked manuscript to enable reviewers easily track the changes made as per their comments.

**Review Comments to the Author**

**Reviewer #3:** The study addresses an important evidence gap and uses valuable routine-care data. However, several concerns regarding exposure definition, missing outcomes, confounding and interpretation require clarification and reanalysis.

**Review comments**

1. Lines 42–49: The missingness of pregnancy outcome data for 49 women is mentioned in the abstract. Clarification on whether multiple imputation was applied only to covariates or also to the outcome.

***Response:*** *Thanks for the comment*

*Multiple imputation was applied on covariates and outcome. The statement on handling missing data has been edited in abstract and methods section. Abstract lines 48 – 49, methods section 248 – 249.*

2. Lines 171–182: Preterm delivery has been removed from the revised abstract and primary composite, but it remains in parts of the objective and the footnote explanation of Tables 2 and 3. Since preterm birth was not reliably documented, it should be removed from all analytical outcome definitions. Consider using “none were documented” rather than “no cases occurred.”

***Response:*** *Thanks for this comment*

*We agree that preterm delivery should not be included as an analytical outcome because it was not reliably documented in the available data sources. Accordingly, preterm delivery has been removed from the revised outcome definitions and from the footnotes of Tables 2 and 3 (Lines 384–386 and 411–412).*

*However, preterm delivery has been retained in the study objective to reflect the standard range of adverse pregnancy outcomes commonly reported in the literature, although, no cases were documented in our dataset. We have revised the manuscript throughout to consistently state that “no cases of preterm delivery were documented” rather than “no cases occurred.”*

*For transparency and completeness, we retained a brief statement in the Results section indicating that no preterm delivery cases were documented in the available data sources, allowing readers to interpret this outcome appropriately.*

3. Lines 178–218: The exposure definition may introduce immortal-time bias. Follow-up begins at the first documented pregnancy visit, but women are classified as exposed if they initiate IPT later during pregnancy. Women must therefore remain pregnant and event-free until IPT initiation to enter the exposed group, while earlier events are assigned to the comparison group. This is not an intention-to-treat analysis. Please consider a time-varying exposure analysis or a prespecified gestational landmark that aligns eligibility, treatment assignment and follow-up. If reanalysis is not feasible, this limitation should be acknowledged and causal interpretations avoided.

***Response:*** *We thank the reviewer for this important observation.*

*We agree that our exposure definition may introduce immortal-time bias because follow-up began at the first antenatal visit, whereas TPT initiation could occur later in pregnancy. We considered a time-varying exposure analysis; however, incomplete dates for pregnancy outcomes in the source records precluded this approach. We have therefore explicitly acknowledged this limitation in the manuscript and revised the text to avoid causal interpretations of the findings. Also, a phrase of “an intention-to-treat analysis” has been removed.*

*The following statement has been added in limitation section (line 492 – 497):*

*“Lastly, immortal-time bias may have occurred because TPT initiation could occur after the first antenatal visit, requiring exposed women to remain free of adverse pregnancy outcomes until treatment initiation. Although a time-varying exposure analysis was not feasible due to incomplete outcome dates, this bias is unlikely to have materially affected our findings, as a secondary analysis showed no significant differences in outcomes between women initiating TPT in the first trimester and those initiating treatment later in pregnancy.”*

4. Lines 209–218 and 273–277: The comparison group should not be described as “TPT-unexposed,” because 149 of 294 women had completed IPT before pregnancy. Can be reframed as “IPT initiation during pregnancy versus no initiation during pregnancy.” A sensitivity analysis restricted to women who had never received IPT, or a three-category analysis separating pregnancy initiation, pre-pregnancy completion and never-exposed women, would be meaningful.

***Response:*** *Thanks for the comment*

We agree that the comparison group should not be interpreted as entirely TPT-unexposed, as some women had completed TPT before pregnancy. However, we retained the classification of “TPT exposed during pregnancy” versus “TPT unexposed during pregnancy” because exposure during pregnancy included both women who initiated TPT while pregnant and those who conceived while still receiving an ongoing TPT course. To improve clarity, we have added the following statement to the Methods section (Lines 207–208):

“*TPT exposed during pregnancy implies that a woman received TPT either as a continuing course or through initiation during pregnancy.*” (lines 207 – 208).

Regarding the suggested sensitivity analysis, the limited number of adverse pregnancy outcomes precluded adequately powered adjusted analyses for additional exposure categories. Nevertheless, we conducted descriptive analyses stratifying women as: never exposed to TPT, completed TPT before pregnancy, and exposed to TPT during pregnancy. These results are presented in Supplementary Table S5 and showed no significant difference in composite adverse pregnancy outcomes across the three groups (p=0.781). We have added the corresponding methods (Lines 211–213) and results statements (Lines 341–344), and updated Supplementary Table S4 to include the composite adverse pregnancy outcome.

5. Lines 220–233: Ensure that all propensity-score covariates were measured before IPT initiation. Viral load was allowed to be measured up to six months after the pregnancy event date and may therefore be post-exposure or post-outcome. The timing of BMI, anaemia and ART regimen should also be clarified. Post-exposure variables should not be treated as baseline confounders.

***Response:*** *Thank you for this important comment.*

*All propensity score covariates were intended to represent baseline characteristics. BMI, anaemia status, and ART regimen were measured at or before the first antenatal care visit and prior to IPT initiation. For viral load, measurements obtained at the first antenatal visit were preferentially used. However, due to infrequent viral load monitoring in routine HIV care, 30 women had no measurement recorded at the first antenatal visit. For these women, the closest viral load measurement within six months of enrolment was used; all were recorded within two weeks of TPT initiation. We therefore considered these values as proxies for baseline viral load. To acknowledge the potential for post-exposure measurement and residual bias, we have added this as a study limitation in the revised manuscript.*

*The following statement has been added under limitation section (lines 489 – 491).*

*“Another limitation was the sparse availability of viral load and CD4 measurements in routine care, necessitating a relaxed baseline assessment window that included measurements obtained up to one week after TPT initiation for some women.”*

6. Lines 235–266: The multiple-imputation and IPTW procedures require detailed reporting. Specify whether propensity scores were estimated separately within each imputed dataset, whether the outcome was imputed, how weighted estimates and standard errors were pooled, and the effective sample size after weighting. The adequacy of 10 imputations should be justified, given 25% missing CD4 and 36% missing viral-load data, and also explain the rationale for using the last observation carried forward for weight.

***Response: Thanks for the comment***

***Specify whether propensity scores were estimated separately within each imputed dataset***

*Yes: propensity scores estimated separately within each imputed dataset. Statement has been included in methods section (lines 258 – 259)*

***whether the outcome was imputed***

*Yes: the outcome was imputed and included in the imputation model. This has been indicated in methods section (lines 248 – 257)*

***how weighted estimates and standard errors were pooled***

*The weighted logistic regression was fitted separately in each imputed dataset and then pooled using: Rubin's rules for combining estimates and standard errors. This is now clearly described in methods section (lines 262 – 264).*

***The effective sample size after weighting***

*Effective sample size after weighting has been estimated and included in methods and results in revised manuscript (methods lines 264 – 269, results lines 344 – 346).*

***The adequacy of 10 imputations should be justified, given 25% missing CD4 and 36% missing viral-load data***

*Ten imputations were used as reasonable start, but number of imputations were also increased to 20 and 30, close to proportion of high missing values in the dataset; to check if it substantially changes. The rationale for choice of 10 imputations has been added in the methods section (lines 249 – 251). A statement on estimates after using various number of imputations (20 and 30 imputations) has been added in results section (lines 355 – 356).*

*On overall, the Methods section has been expanded to provide additional details on the multiple-imputation and IPTW procedures (method section, lines 248 – 269).*

***Explain the rationale for using the last observation carried forward for weight.***

*Baseline weight and selected continuous covariates were occasionally missing at study baseline despite participants having measurements recorded at earlier clinic visits. In these instances, we used the most recent prior recorded value (last observation carried forward, LOCF) to populate the missing baseline value. This approach was chosen because these variables represented baseline characteristics rather than longitudinal outcomes, and prior measurements obtained close to baseline were considered more informative than excluding participants or relying on model-based imputation for a small amount of missing data. We have clarified this rationale in the Methods section (lines 237 – 242).*

*We acknowledge that LOCF may not fully reflect changes occurring between the previous measurement and baseline, and may underestimate variability; however, the proportion of missing baseline covariate data requiring LOCF was small.*

7. Lines 304–327: Pregnancy outcomes were missing for 6.2% of women initiating IPT during pregnancy and 11.9% of the comparison group. A comparison of baseline characteristics among participants with and without observed outcomes by exposure group would provide a better understanding.

***Response****: Thanks for the comment*

*Summary on comparison of missingness on outcome across baseline characteristics by exposure groups has been added (supplementary Table S7). There was no significant difference between observed and unobserved outcomes across baseline characteristics.*

*The following statement has also been added in results section (lines 329 – 330).*

*“No significant differences between observed and unobserved baseline characteristics (supplementary Table S7).”*

8. Lines 349–364: For sparse individual outcomes and zero cells, Fisher’s exact test would be more appropriate than Pearson’s chi-square test.

***Response****: Thanks for the comment*

*Fisher’s exact p-values have been added for table cells with sparse count, i.e. where the expected counts are less than 5.*
