# Supplementary material for: Isoniazid monotherapy for tuberculosis prevention during pregnancy and adverse outcomes in women living with HIV in Uganda: A propensity score–weighted analysis of routine care data
Source: PLOS Glob Public Health. 2026 Sep 25;6(9):e0005829. doi: 10.1371/journal.pgph.0005829 (PMC13614580; doi:10.1371/journal.pgph.0005829)
Supplement: Peer review history

# Peer review: Decision Letter 0

**DOI:** 10.1371/journal.pgph.0005829.r001

**Authors:** Collins Asweto

17 Dec 2025

PGPH-D-25-03149

Impact of tuberculosis preventive treatment on adverse pregnancy outcomes in women living with HIV in Uganda:  an inverse probability for treatment weighting analysis of routine care data

PLOS Global Public Health

Dear Musaazi,

Thank you for submitting your manuscript to PLOS Global Public Health. After careful consideration, we feel that it has merit but does not fully meet PLOS Global Public Health’s publication criteria as it currently stands. Therefore, we invite you to submit a revised version of the manuscript that addresses the points raised during the review process.

Please submit your revised manuscript by 16th February, 2026. If you will need more time than this to complete your revisions, please reply to this message or contact the journal office at globalpubhealth@plos.org. When you're ready to submit your revision, log on to https://www.editorialmanager.com/pgph/ and select the 'Submissions Needing Revision' folder to locate your manuscript file.

Please include the following items when submitting your revised manuscript:

- A letter that responds to each point raised by the editor and reviewer(s). You should upload this letter as a separate file labeled 'Response to Reviewers'.
- A marked-up copy of your manuscript that highlights changes made to the original version. You should upload this as a separate file labeled 'Revised Manuscript with Track Changes'.
- An unmarked version of your revised paper without tracked changes. You should upload this as a separate file labeled 'Manuscript'.

Guidelines for resubmitting your figure files are available below the reviewer comments at the end of this letter.

We look forward to receiving your revised manuscript.

Kind regards,

Collins Otieno Asweto, PhD

Academic Editor

PLOS Global Public Health

Journal Requirements:

If the reviewer comments include a recommendation to cite specific previously published works, please review and evaluate these publications to determine whether they are relevant and should be cited. There is no requirement to cite these works unless the editor has indicated otherwise.

Please review your reference list to ensure that it is complete and correct. If you have cited papers that have been retracted, please include the rationale for doing so in the manuscript text, or remove these references and replace them with relevant current references. Any changes to the reference list should be mentioned in the rebuttal letter that accompanies your revised manuscript. If you need to cite a retracted article, indicate the article’s retracted status in the References list and also include a citation and full reference for the retraction notice.

Additional Editor Comments (if provided):

[Note: HTML markup is below. Please do not edit.]

Reviewers' comments:

Reviewer's Responses to Questions

**Comments to the Author**

1. Does this manuscript meet PLOS Global Public Health’s publication criteria?

Reviewer #1: Yes

Reviewer #2: Yes

**********

2. Has the statistical analysis been performed appropriately and rigorously?

Reviewer #1: Yes

Reviewer #2: Yes

**********

3. Have the authors made all data underlying the findings in their manuscript fully available (please refer to the Data Availability Statement at the start of the manuscript PDF file)?

The PLOS Data policy

Reviewer #1: Yes

Reviewer #2: Yes

**********

4. Is the manuscript presented in an intelligible fashion and written in standard English?

Reviewer #1: Yes

Reviewer #2: Yes

**********

Reviewer #1: Review report

L1, L96: Title, objective: be consistent with the words you use: Impact??? Association? The use of “estimated effect” could be scientifically sound.

Abstract: Make a structured abstract

L63-L70: Very long sentences. Don’t exceed 25 for one sentence. Check the whole study.

L128-145: Relocate these paragraphs in introduction as they explain the program. Instead, it mentions what was based on from this program to select the study participants or data to include in this study.

L153: A total sample size of 538 participants. How have you found this sample size. By which method or sample size formula have you used to get this sample size. Could you mention the study population number. This means all the population that were found the 5 selected health facilities in Kampala.

L238-L243:

o Relocate this. This should be written after the population study before sampling procedure. Because it helps to understand the flow and digestibility of information. Eg. Study design- sample-selection criteria and so on.

o Make sure to reference the baseline risks for ensuring that they are evidenced-based not arbitrary. Because 17% baseline risk in the unexposed group will influence the results and the conclusion.

o Explain in few words in sample size. How you have assigned the exposed and non-exposed sample.

o There is a lack of summarized protocol of information flow from study population number- exclusion number -total sample number and group sample number.

o Mention the software that you have used.

382-Discussion

o Discuss the use of small sample size with more missing data and non-compliance of IPT through out the 3 trimesters.

o Discuss this study implications.

Reviewer #2: The study uses an appropriate causal-inference approach (IPTW with multiple imputation) to assess the safety of IPT during pregnancy using routine data. While relevant confounders were identified and included, the validity of IPTW-adjusted estimates depends on demonstrating that the method successfully balanced these covariates. Because the manuscript does not present any balance diagnostics, it remains unclear whether confounding was adequately controlled.

The authors should report IPTW balance diagnostics, including standardized mean differences before and after weighting, and the distribution of stabilized weights. These diagnostics are essential to confirm that the IPTW model effectively reduced confounding using the available data. If it is not possible, the authors should explicitely mention this as limitation

**********

what does this mean?). If published, this will include your full peer review and any attached files.

**Do you want your identity to be public for this peer review?**  If you choose “no”, your identity will remain anonymous but your review may still be made public.

For information about this choice, including consent withdrawal, please see our Privacy Policy

Reviewer #1: **Yes:** Nsanzabera Charles

Reviewer #2: No

**********

[NOTE: If reviewer comments were submitted as an attachment file, they will be attached to this email and accessible via the submission site. Please log into your account, locate the manuscript record, and check for the action link "View Attachments". If this link does not appear, there are no attachment files.]

Attachment Submitted filename: Review report.docx

# Author comment: Author response to Decision Letter 1

**DOI:** 10.1371/journal.pgph.0005829.r002

30 Dec 2025

Attachment Submitted filename: Response to Reviewers.docx

# Peer review: Decision Letter 1

**DOI:** 10.1371/journal.pgph.0005829.r003

**Authors:** Alice Zwerling

30 Apr 2026

PGPH-D-25-03149R1

Effect of tuberculosis preventive treatment on adverse pregnancy outcomes in women living with HIV in Uganda:  an inverse probability for treatment weighting analysis of routine care data

PLOS Global Public Health

Dear Dr. Musaazi,

Thank you for submitting your manuscript to PLOS Global Public Health. After careful consideration, we feel that it has merit but does not fully meet PLOS Global Public Health’s publication criteria as it currently stands. Therefore, we invite you to submit a revised version of the manuscript that addresses the points raised during the review process.

Please submit your revised manuscript by May 30 2026 11:59PM. If you will need more time than this to complete your revisions, please reply to this message or contact the journal office at globalpubhealth@plos.org. When you're ready to submit your revision, log on to https://www.editorialmanager.com/pgph/ and select the 'Submissions Needing Revision' folder to locate your manuscript file.

Please include the following items when submitting your revised manuscript:

- A letter that responds to each point raised by the editor and reviewer(s). You should upload this letter as a separate file labeled 'Response to Reviewers'.
- A marked-up copy of your manuscript that highlights changes made to the original version. You should upload this as a separate file labeled 'Revised Manuscript with Track Changes'.
- An unmarked version of your revised paper without tracked changes. You should upload this as a separate file labeled 'Manuscript'.

Guidelines for resubmitting your figure files are available below the reviewer comments at the end of this letter.

As the corresponding author, your ORCID iD is verified in the submission system and will appear in the published article. PLOS supports the use of ORCID, and we encourage all coauthors to register for an ORCID iD and use it as well. Please encourage your coauthors to verify their ORCID iD within the submission system before final acceptance, as unverified ORCID iDs will not appear in the published article. *Only*  the individual author can complete the verification step; PLOS staff *cannot*  verify ORCID iDs on behalf of authors.

We look forward to receiving your revised manuscript.

Kind regards,

Alice Zwerling, PhD

Academic Editor

PLOS Global Public Health

**Journal Requirements:**

If the reviewer comments include a recommendation to cite specific previously published works, please review and evaluate these publications to determine whether they are relevant and should be cited. There is no requirement to cite these works unless the editor has indicated otherwise.

Please review your reference list to ensure that it is complete and correct. If you have cited papers that have been retracted, please include the rationale for doing so in the manuscript text, or remove these references and replace them with relevant current references. Any changes to the reference list should be mentioned in the rebuttal letter that accompanies your revised manuscript. If you need to cite a retracted article, indicate the article’s retracted status in the References list and also include a citation and full reference for the retraction notice.

**Additional Editor Comments (if provided):**

[Note: HTML markup is below. Please do not edit.]

Reviewers' comments:

Reviewer's Responses to Questions

**Comments to the Author**

Reviewer #2: All comments have been addressed

**********

publication criteria?

Reviewer #2: Yes

**********

3. Has the statistical analysis been performed appropriately and rigorously?

Reviewer #2: Yes

**********

4. Have the authors made all data underlying the findings in their manuscript fully available (please refer to the Data Availability Statement at the start of the manuscript PDF file)?

The PLOS Data policy

Reviewer #2: Yes

**********

5. Is the manuscript presented in an intelligible fashion and written in standard English?

Reviewer #2: Yes

**********

**Reviewer #2:**  Thank you for the revision. While IPTW with multiple imputation is appropriate, the manuscript still does not present balance diagnostics to demonstrate that weighting adequately controlled confounding. Please report standardized mean differences before and after weighting and describe the distribution of stabilized weights (or acknowledge this as a limitation if unavailable). In addition, as this is an observational study, please state that reporting follows STROBE guidelines and include the completed STROBE checklist in the supplementary material. Finally, please review the manuscript carefully for minor grammatical and wording errors before final submission.

**********

what does this mean?). If published, this will include your full peer review and any attached files.

**Do you want your identity to be public for this peer review?**  If you choose “no”, your identity will remain anonymous but your review may still be made public.

For information about this choice, including consent withdrawal, please see our Privacy Policy

Reviewer #2: No

**********

[NOTE: If reviewer comments were submitted as an attachment file, they will be attached to this email and accessible via the submission site. Please log into your account, locate the manuscript record, and check for the action link "View Attachments". If this link does not appear, there are no attachment files.]

**Figure Resubmissions:**

After uploading your figures to PLOS’s NAAS tool - https://ngplosjournals.pagemajik.ai/artanalysis, NAAS will process the files provided and display the results in the "Uploaded Files" section of the page as the processing is complete. If the uploaded figures meet our requirements (or NAAS is able to fix the files to meet our requirements), the figure will be marked as "fixed" above. If NAAS is unable to fix the files, a red "failed" label will appear above. When NAAS has confirmed that the figure files meet our requirements, please download the file via the download option, and include these NAAS processed figure files when submitting your revised manuscript.

# Author comment: Author response to Decision Letter 2

**DOI:** 10.1371/journal.pgph.0005829.r004

2 May 2026

Attachment Submitted filename: Response_to_Reviewers_auresp_2.docx

# Peer review: Decision Letter 2

**DOI:** 10.1371/journal.pgph.0005829.r005

**Authors:** Alice Zwerling

17 May 2026

PGPH-D-25-03149R2

Effect of tuberculosis preventive treatment on adverse pregnancy outcomes in women living with HIV in Uganda:  an inverse probability for treatment weighting analysis of routine care data

PLOS Global Public Health

Dear Dr. Musaazi,

Thank you for submitting your manuscript to PLOS Global Public Health. After careful consideration, we feel that it has merit but does not fully meet PLOS Global Public Health’s publication criteria as it currently stands. Therefore, we invite you to submit a revised version of the manuscript that addresses the points raised during the review process.

Please submit your revised manuscript by Jun 16 2026 11:59PM. If you will need more time than this to complete your revisions, please reply to this message or contact the journal office at globalpubhealth@plos.org. When you're ready to submit your revision, log on to https://www.editorialmanager.com/pgph/ and select the 'Submissions Needing Revision' folder to locate your manuscript file.

Please include the following items when submitting your revised manuscript:

- A letter that responds to each point raised by the editor and reviewer(s). You should upload this letter as a separate file labeled 'Response to Reviewers'.
- A marked-up copy of your manuscript that highlights changes made to the original version. You should upload this as a separate file labeled 'Revised Manuscript with Track Changes'.
- An unmarked version of your revised paper without tracked changes. You should upload this as a separate file labeled 'Manuscript'.

Guidelines for resubmitting your figure files are available below the reviewer comments at the end of this letter.

As the corresponding author, your ORCID iD is verified in the submission system and will appear in the published article. PLOS supports the use of ORCID, and we encourage all coauthors to register for an ORCID iD and use it as well. Please encourage your coauthors to verify their ORCID iD within the submission system before final acceptance, as unverified ORCID iDs will not appear in the published article. *Only*  the individual author can complete the verification step; PLOS staff *cannot*  verify ORCID iDs on behalf of authors.

We look forward to receiving your revised manuscript.

Kind regards,

Alice Zwerling, PhD

Academic Editor

PLOS Global Public Health

**Journal Requirements:**

If the reviewer comments include a recommendation to cite specific previously published works, please review and evaluate these publications to determine whether they are relevant and should be cited. There is no requirement to cite these works unless the editor has indicated otherwise.

Please review your reference list to ensure that it is complete and correct. If you have cited papers that have been retracted, please include the rationale for doing so in the manuscript text, or remove these references and replace them with relevant current references. Any changes to the reference list should be mentioned in the rebuttal letter that accompanies your revised manuscript. If you need to cite a retracted article, indicate the article’s retracted status in the References list and also include a citation and full reference for the retraction notice.

**Additional Editor Comments (if provided):**

Thanks for your attention to the previous reviewer comments, there are only a few minor revisions requested at this stage.

[Note: HTML markup is below. Please do not edit.]

Reviewers' comments:

Reviewer's Responses to Questions

**Comments to the Author**

Reviewer #2: All comments have been addressed

**********

publication criteria?

Reviewer #2: Yes

**********

3. Has the statistical analysis been performed appropriately and rigorously?

Reviewer #2: Yes

**********

4. Have the authors made all data underlying the findings in their manuscript fully available (please refer to the Data Availability Statement at the start of the manuscript PDF file)?

The PLOS Data policy

Reviewer #2: No

**********

5. Is the manuscript presented in an intelligible fashion and written in standard English?

Reviewer #2: Yes

**********

**Reviewer #2:**  The manuscript has improved substantially and the methodology is presented more clearly in this revised version. The use of IPTW, sensitivity analyses, and acknowledgement of limitations strengthen the paper. I only have a few minor comments remaining before publication consideration.

The manuscript still occasionally uses causal language such as “effect,” “impact,” “risk,” and “causal inference models” despite the observational retrospective design. Consider replacing these with more cautious terms such as “association,” “odds,” or “propensity score weighted analysis.”

There remains a reporting inconsistency regarding preterm delivery. The abstract and Table 2 include preterm delivery in the composite outcome definition, whereas the Methods section states that preterm birth was excluded due to lack of documentation. This should be harmonized throughout the manuscript.

In Table 1, viral load percentages appear to be calculated among participants with available viral load measurements rather than the full cohort. Since 36% of viral load data were missing, the denominators should be clarified in the table footnote to avoid misinterpretation.

The category “Other anomalies: Difficulty in breathing” in Table 2 would benefit from clarification, as difficulty in breathing may represent a neonatal complication rather than a congenital anomaly.

The subgroup and trimester analyses are interesting, but the very small number of events should be emphasized more clearly when interpreting these findings, particularly regarding stillbirths.

**********

what does this mean?). If published, this will include your full peer review and any attached files.

**Do you want your identity to be public for this peer review?**  If you choose “no”, your identity will remain anonymous but your review may still be made public.

For information about this choice, including consent withdrawal, please see our Privacy Policy

Reviewer #2: No

**********

[NOTE: If reviewer comments were submitted as an attachment file, they will be attached to this email and accessible via the submission site. Please log into your account, locate the manuscript record, and check for the action link "View Attachments". If this link does not appear, there are no attachment files.]

**Figure Resubmissions:**

# Author comment: Author response to Decision Letter 3

**DOI:** 10.1371/journal.pgph.0005829.r006

18 May 2026

Attachment Submitted filename: Response to Reviewers 18May2026.docx

# Peer review: Decision Letter 3

**DOI:** 10.1371/journal.pgph.0005829.r007

**Authors:** Sonali Sarkar

4 Aug 2026

PGPH-D-25-03149R3

Effect of tuberculosis preventive treatment on adverse pregnancy outcomes in women living with HIV in Uganda:  an inverse probability for treatment weighting analysis of routine care data

PLOS Global Public Health

Dear Dr. Musaazi,

Thank you for submitting your manuscript to PLOS Global Public Health. After careful consideration, we feel that it has merit but does not fully meet PLOS Global Public Health’s publication criteria as it currently stands. Therefore, we invite you to submit a revised version of the manuscript that addresses the points raised during the review process.

Appropriate methods have been used to answer a pertinent question using routinely collecyed data. However, multiple clarifications are required in the process adopted and therefore a more detailed reporting of the methods is necessary. Though one of the reviewers suggested 'major revision', it is assumed that these processes have been conducted and a re-analysis may not be necessary.

Please submit your revised manuscript by Sep 03 2026 11:59PM. If you will need more time than this to complete your revisions, please reply to this message or contact the journal office at globalpubhealth@plos.org. When you're ready to submit your revision, log on to https://www.editorialmanager.com/pgph/ and select the 'Submissions Needing Revision' folder to locate your manuscript file.

Please include the following items when submitting your revised manuscript:

- A letter that responds to each point raised by the editor and reviewer(s). You should upload this letter as a separate file labeled 'Response to Reviewers'.
- A marked-up copy of your manuscript that highlights changes made to the original version. You should upload this as a separate file labeled 'Revised Manuscript with Track Changes'.
- An unmarked version of your revised paper without tracked changes. You should upload this as a separate file labeled 'Manuscript'.

Guidelines for resubmitting your figure files are available below the reviewer comments at the end of this letter.

As the corresponding author, your ORCID iD is verified in the submission system and will appear in the published article. PLOS supports the use of ORCID, and we encourage all coauthors to register for an ORCID iD and use it as well. Please encourage your coauthors to verify their ORCID iD within the submission system before final acceptance, as unverified ORCID iDs will not appear in the published article. *Only*  the individual author can complete the verification step; PLOS staff *cannot*  verify ORCID iDs on behalf of authors.

We look forward to receiving your revised manuscript.

Kind regards,

Sonali Sarkar

Academic Editor

PLOS Global Public Health

Journal Requirements:

If the reviewer comments include a recommendation to cite specific previously published works, please review and evaluate these publications to determine whether they are relevant and should be cited. There is no requirement to cite these works unless the editor has indicated otherwise.

Please review your reference list to ensure that it is complete and correct. If you have cited papers that have been retracted, please include the rationale for doing so in the manuscript text, or remove these references and replace them with relevant current references. Any changes to the reference list should be mentioned in the rebuttal letter that accompanies your revised manuscript. If you need to cite a retracted article, indicate the article’s retracted status in the References list and also include a citation and full reference for the retraction notice.

Additional Editor Comments (if provided):

Please respond to the queries from the reviewer 3

[Note: HTML markup is below. Please do not edit.]

Reviewers' comments:

Reviewer's Responses to Questions

**Comments to the Author**

Reviewer #3: (No Response)

Reviewer #4: All comments have been addressed

**********

publication criteria?

Reviewer #3: Yes

Reviewer #4: Partly

**********

3. Has the statistical analysis been performed appropriately and rigorously?

Reviewer #3: Yes

Reviewer #4: No

**********

4. Have the authors made all data underlying the findings in their manuscript fully available (please refer to the Data Availability Statement at the start of the manuscript PDF file)?

The PLOS Data policy

Reviewer #3: Yes

Reviewer #4: (No Response)

**********

5. Is the manuscript presented in an intelligible fashion and written in standard English?

Reviewer #3: Yes

Reviewer #4: Yes

**********

Reviewer #3: The study addresses an important evidence gap and uses valuable routine-care data. However, several concerns regarding exposure definition, missing outcomes, confounding and interpretation require clarification and reanalysis.

Review comments

1. Lines 42–49: The missingness of pregnancy outcome data for 49 women is mentioned in the abstract. Clarification on whether multiple imputation was applied only to covariates or also to the outcome.

2. Lines 171–182: Preterm delivery has been removed from the revised abstract and primary composite, but it remains in parts of the objective and the footnote explanation of Tables 2 and 3. Since preterm birth was not reliably documented, it should be removed from all analytical outcome definitions. Consider using “none were documented” rather than “no cases occurred.”

3. Lines 178–218: The exposure definition may introduce immortal-time bias. Follow-up begins at the first documented pregnancy visit, but women are classified as exposed if they initiate IPT later during pregnancy. Women must therefore remain pregnant and event-free until IPT initiation to enter the exposed group, while earlier events are assigned to the comparison group. This is not an intention-to-treat analysis. Please consider a time-varying exposure analysis or a prespecified gestational landmark that aligns eligibility, treatment assignment and follow-up. If reanalysis is not feasible, this limitation should be acknowledged and causal interpretations avoided.

4. Lines 209–218 and 273–277: The comparison group should not be described as “TPT-unexposed,” because 149 of 294 women had completed IPT before pregnancy. Can be reframed as “IPT initiation during pregnancy versus no initiation during pregnancy.” A sensitivity analysis restricted to women who had never received IPT, or a three-category analysis separating pregnancy initiation, pre-pregnancy completion and never-exposed women, would be meaningful.

5. Lines 220–233: Ensure that all propensity-score covariates were measured before IPT initiation. Viral load was allowed to be measured up to six months after the pregnancy event date and may therefore be post-exposure or post-outcome. The timing of BMI, anaemia and ART regimen should also be clarified. Post-exposure variables should not be treated as baseline confounders.

6. Lines 235–266: The multiple-imputation and IPTW procedures require detailed reporting. Specify whether propensity scores were estimated separately within each imputed dataset, whether the outcome was imputed, how weighted estimates and standard errors were pooled, and the effective sample size after weighting. The adequacy of 10 imputations should be justified, given 25% missing CD4 and 36% missing viral-load data, and also explain the rationale for using the last observation carried forward for weight.

7. Lines 304–327: Pregnancy outcomes were missing for 6.2% of women initiating IPT during pregnancy and 11.9% of the comparison group. A comparison of baseline characteristics among participants with and without observed outcomes by exposure group would provide a better understanding.

8. Lines 349–364: For sparse individual outcomes and zero cells, Fisher’s exact test would be more appropriate than Pearson’s chi-square test.

Reviewer #4: The Methdology component of the article needed explained in detail for better understanding.

**********

what does this mean?). If published, this will include your full peer review and any attached files.

**Do you want your identity to be public for this peer review?**  If you choose “no”, your identity will remain anonymous but your review may still be made public.

For information about this choice, including consent withdrawal, please see our Privacy Policy

Reviewer #3: **Yes:** Vignesh Rajan S

Reviewer #4: No

**********

[NOTE: If reviewer comments were submitted as an attachment file, they will be attached to this email and accessible via the submission site. Please log into your account, locate the manuscript record, and check for the action link "View Attachments". If this link does not appear, there are no attachment files.]

Attachment Submitted filename: Comments.docx

Attachment Submitted filename: PGPH-D-25-03149R3_ comments.pdf

# Author comment: Author response to Decision Letter 4

**DOI:** 10.1371/journal.pgph.0005829.r008

10 Aug 2026

Attachment Submitted filename: Response_to_Reviewers_auresp_4.docx

# Editor report: Decision Letter 4

**DOI:** 10.1371/journal.pgph.0005829.r009

**Authors:** Sonali Sarkar

26 Aug 2026

Isoniazid monotherapy for tuberculosis prevention during pregnancy and adverse outcomes in women living with HIV in Uganda:  A Propensity Score–Weighted Analysis of Routine Care Data

PGPH-D-25-03149R4

Dear Dr Musaazi,

We are pleased to inform you that your manuscript 'Isoniazid monotherapy for tuberculosis prevention during pregnancy and adverse outcomes in women living with HIV in Uganda:  A Propensity Score–Weighted Analysis of Routine Care Data' has been provisionally accepted for publication in PLOS Global Public Health.

Before your manuscript can be formally accepted you will need to complete some formatting changes, which you will receive in a follow up email. A member of our team will be in touch with a set of requests.

Please note that your manuscript will not be scheduled for publication until you have made the required changes, so a swift response is appreciated.

IMPORTANT: The editorial review process is now complete. PLOS will only permit corrections to spelling, formatting or significant scientific errors from this point onwards. Requests for major changes, or any which affect the scientific understanding of your work, will cause delays to the publication date of your manuscript.

If your institution or institutions have a press office, please notify them about your upcoming paper to help maximize its impact. If they'll be preparing press materials, please inform our press team as soon as possible -- no later than 48 hours after receiving the formal acceptance. Your manuscript will remain under strict press embargo until 2 pm Eastern Time on the date of publication. For more information, please contact globalpubhealth@plos.org.

Thank you again for supporting Open Access publishing; we are looking forward to publishing your work in PLOS Global Public Health.

Best regards,

Sonali Sarkar

Academic Editor

PLOS Global Public Health

***********************************************************

Reviewer Comments (if any, and for reference):
